# Jasmonate induced alternative splicing responses in *Arabidopsis*

**DOI:** 10.1002/pld3.245

**Published:** 2020-08-26

**Authors:** Guanqiao Feng, Mi‐Jeong Yoo, Ruth Davenport, J. Lucas Boatwright, Jin Koh, Sixue Chen, W. Brad Barbazuk

**Affiliations:** ^1^ Plant Molecular and Cellular Biology Program University of Florida Gainesville FL USA; ^2^ Department of Biology University of Florida Gainesville FL USA; ^3^ The Interdisciplinary Center for Biotechnology Research (ICBR) University of Florida Gainesville FL USA; ^4^ The Genetics Institute University of Florida Gainesville FL USA

**Keywords:** alternative splicing, gene network, jasmonate signaling, miRNA, splicing factor, transcription factor

## Abstract

Jasmonate is an essential phytohormone regulating plant growth, development, and defense. Alternative splicing (AS) in jasmonate ZIM‐domain (JAZ) repressors is well‐characterized and plays an important role in jasmonate signaling regulation. However, it is unknown whether other genes in the jasmonate signaling pathway are regulated by AS. We explore the potential for AS regulation in three *Arabidopsis* genotypes (WT, *jaz2*, *jaz7*) in response to methyl jasmonate (MeJA) treatment with respect to: (a) differential AS, (b) differential miRNA targeted AS, and (c) AS isoforms with novel functions. AS events identified from transcriptomic data were validated with proteomic data. Protein interaction networks identified two genes, *SKIP* and *ALY4* whose products have both DNA‐ and RNA‐binding affinities, as potential key regulators mediating jasmonate signaling and AS regulation. We observed cases where AS alone, or AS and transcriptional regulation together, can influence gene expression in response to MeJA. Twenty‐one genes contain predicted miRNA target sites subjected to AS, which implies that AS is coupled to miRNA regulation. We identified 30 cases where alternatively spliced isoforms may have novel functions. For example, AS of *bHLH160* generates an isoform without a basic domain, which may convert it from an activator to a repressor. Our study identified potential key regulators in AS regulation of jasmonate signaling pathway. These findings highlight the importance of AS regulation in the jasmonate signaling pathway, both alone and in collaboration with other regulators.

**Significance statement:**

By exploring alternative splicing, we demonstrate its regulation in the jasmonate signaling pathway alone or in collaboration with other posttranscriptional regulations such as nonsense and microRNA‐mediated decay. A signal transduction network model for alternative splicing in jasmonate signaling pathway was generated, contributing to our understanding for this important, prevalent, but relatively unexplored regulatory mechanism in plants.

## 1 INTRODUCTION

Plants have developed inducible defense mechanisms and complex signaling networks for efficient, precise and fast response to ever‐changing environmental stimuli and stresses. Jasmonate is a phytohormone induced by herbivore or pathogen attack, as well as abiotic stresses such as cold, salt, UV light, and ozone (Browse & Howe, 2008; Howe & Jander, 2008; Hu, Jiang, Wang, & Yu, 2013; Katsir, Chung, Koo, & Howe, 2008; Valenzuela et al., 2016). Jasmonate regulates a variety of plant events including photosynthesis (Attaran et al., 2014), root and shoot morphogenesis (Gasperini et al., 2015; Zheng et al., 2017), flowering time (Zhai et al., 2015; Thatcher, Danilevskaya, et al., 2016), stamen development (Qi, Huang, Song, & Xie, 2015a; Song et al., 2011), seed germination (Linkies & Leubner‐Metzger, 2012), and senescence (Jiang, Liang, Yang, & Yu, 2014; Miao & Zentgraf, 2007; Qi, Wang, et al., 2015; Yu et al., 2016). In general, jasmonate inhibits plant growth, promotes growth to defense transition and triggers early reproduction.

Biologically active (+)‐7‐*iso*‐Jasmonoyl‐L‐isoleucine (JA‐Ile), Jasmonate ZIM‐domain (JAZ) proteins and the F‐box protein of the E3 ubiquitin‐ligase complex Coronatine Insensitive 1 (COI1) form the key three component regulatory module mediating jasmonate signal transduction (Chini et al., 2007; Fonseca et al., 2009; Thines et al., 2007). Upon JA‐Ile accumulation, the three‐component module (JA‐Ile, COI1 and JAZ) is formed, and the JAZs are polyubiquitinated and degraded through the 26S proteasome, which releases suppression of the JAZ‐interacting transcription factors that regulate early‐jasmonate‐responsive genes (Chini et al., 2007; Chini, Gimenez‐Ibanez, Goossens, & Solano, 2016; Thines et al., 2007). Thirteen JAZ genes have been identified in the *Arabidopsis* genome (Bai, Meng, Huang, Qi, & Chen, 2011; Thireault et al., 2015), and they have both functional redundancy and specificity (Chini et al., 2016). Members of the JAZ family share a conserved N‐terminal domain (Moreno et al., 2013), a TIFY (previously known as ZIM) domain (except JAZ13), and a C‐terminal Jas domain (Chini et al., 2007; Thines et al., 2007; Yan et al., 2007). The TIFY domain is responsible for homo‐ and hetero‐dimerization (Chini, Fonseca, Chico, Fernández‐Calvo, & Solano, 2009) and interaction with NOVEL INTERACTOR OF JAZ (NINJA), which further recruits the co‐repressor TOPLESS (TPL) through its conserved ERF‐associated amphiphilic repression (EAR) domain (Pauwels et al., 2010). However, JAZ5, JAZ6, JAZ7, JAZ8, and JAZ13 could directly recruit TPL by EAR in their N‐terminal domain (Causier, Ashworth, Guo, & Davies, 2012; Shyu et al., 2012; Thireault et al., 2015; Thatcher, Cevik, et al., 2016). The Jas domain is the major domain that is responsible for interaction with transcription factors (Chini et al., 2016), and COI1, except in JAZ7 and JAZ8 where the Jas domain has diverged (Chini et al., 2016; Shyu et al., 2012). Interestingly, almost all the JAZs (except for JAZ1, JAZ7, and JAZ8) share a homologous intron (Jas intron) that divides the Jas domain into a 20‐amino acid N‐terminal motif and a 7‐amino acid C‐terminal motif (X_5_PY; Chung et al., 2010). AS around this conserved intron can lead to a truncated protein lacking the X_5_PY motif or lacking the entire Jas domain (Chung et al., 2010; Chung & Howe, 2009; Monte et al., 2019; Moreno et al., 2013; Yan et al., 2007). These truncated proteins retain their ability to interact with transcription factors through the 20‐amino acid N‐terminal motif, or a similar sequence in their N‐terminal domain, but have reduced (or abolished) ability of being recognized by COI1 (Chung & Howe, 2009; Chung et al., 2010; Moreno et al., 2013; Zhang, Ke, et al., 2017). As a result, these AS isoforms avoid degradation and function as dominant repressors in the presence of jasmonate. AS events around the Jas intron are conserved among monocots, eudicots, and bryohpytes, underscoring their functional importance (Chung et al., 2010; Monte et al., 2019).

It was predicted that ~60% of *Arabidopsis* protein‐coding genes are alternatively spliced (Zhang, Calixto, et al., 2017). However, other than JAZ repressors, little is known about AS regulation in the jasmonate signaling pathway. AS generates multiple transcripts from a single gene by regulated differential selection of splice sites. This process is regulated in part by interactions between *cis*‐elements (intronic/exonic splicing enhancer/silencer) and *trans*‐factors (e.g. Serine/Arginine‐rich (SR) splicing activators, heterogeneous nuclear ribonucleoprotein (hnRNP) splicing repressors, spliceosome components, and additional RNA binding factors; Kornblihtt et al., 2013; Meyer, Koester, & Staiger, 2015). Regulation of AS influences the relative proportion of different isoforms for a given gene. Moreover, AS interacts with other regulatory mechanisms such as non‐sense mediated decay (NMD; Kalyna et al., 2012; Kervestin & Jacobson, 2012) and microRNA (miRNA) regulation (Reddy, Marquez, Kalyna, & Barta, 2013). AS frequently results in a pre‐mature termination codon (PTC; Kalyna et al., 2012) that can mark the transcript for degradation through the NMD pathway (Kervestin & Jacobson, 2012). Thus, NMD coupled with AS can function as an important posttranscriptional mechanism to regulate protein levels (Kervestin & Jacobson, 2012). Inclusion/exclusion of the alternative region may introduce/remove miRNA targeting sites within one isoform relative to another (Reddy et al., 2013), thus genes producing AS isoforms may be subject to both AS and miRNA regulation. Finally, if the AS isoform can make a protein product, it may generate the same protein or a different protein depending on whether AS occurs within the untranslated region (UTR) or coding sequence (CDS). Generation of different protein products may result in nonfunctional, partially‐functional, redundantly‐functional, or neo‐functional alterations (Reddy et al., 2013; Staiger & Brown, 2013). Isoforms with partial‐ or neo‐functions are especially interesting as they may lead to different gene functions, as is the case for JAZs (Chung et al., 2010; Chung & Howe, 2009; Moreno et al., 2013; Yan et al., 2007).

Here we integrated transcriptomics and proteomics of WT, *jaz2* and *jaz7* mutant *Arabidopsis* in response to methyl jasmonate (MeJA) to identify three aspects of AS‐dependent regulation potentially impacting the jasmonate signaling pathway: (a) differential AS in response to MeJA treatment; (b) miRNA regulation edited by differential use of AS isoforms; and (c) AS splice variants with novel functions. The *jaz2* and *jaz7* mutants are within genes that are in different subgroups of JAZ family (Chung et al., 2010). In addition, *jaz2* is a knock‐down mutant and *jaz7* is an over‐expression mutant, both of which exhibit obvious phenotypic changes compared to WT (Yan et al., 2014). RNA‐Seq analysis of the *jaz2* and *jaz7* mutants have been described previously (Yan et al., 2014) and the underlying sequence data are available in the NCBI short‐read archive (SRP026541). We re‐analyzed these data to discover AS isoforms and examine these for differential expression under MeJA treatment—many of which we further explored and describe herein. The AS events identified from the RNA‐seq data were further validated with previously unpublished proteomics data that was generated from the same samples used by Yan et al. (2014) for the RNA‐Seq data acquisition.

## 2 EXPERIMENTAL PROCEDURES

### 2.1 Experimental design and transcriptome/proteome library preparation and sequencing

Thirty‐six RNA‐seq samples corresponding to three replicates each from shoots and roots obtained from the *Arabidopsis thaliana* mutants *jaz2* (SALK079895C) and *jaz7* (SALK040835C) and WT (Col‐0) with (10 μM) or without (0 μM) MeJA treatment were used for transcriptome and proteome profiling. The transcriptome data were published (Yan et al., 2014) and available in the NCBI Sequence Read Archive under SRP026541. The proteome data that were newly generated have been deposited in the ProteomeXchange MassIVE partner repository with the data set identifiers PXD014563 and MSV000084071.

### 2.2 Transcriptome assembly and differential AS detection

Raw single end reads (75 bp) obtained from an Illumina Hiseq2000 instrument were filtered with FASTX‐Toolkit (http://hannonlab.cshl.edu/fastx_toolkit/index.html) and mapped to the *Arabidopsis* reference sequence and annotation (ver. TAIR10 http://www.arabidopsis.org) with GSNAP v2013‐07‐20 (Wu & Nacu, 2010) allowing a maximum intron size of 8,000, a minimum intron size of 20, and a maximum of 5% mismatch. The uniquely mapped reads from each library were assembled into transcripts with Cufflinks v2.2.1 (Trapnell et al., 2013) using the reference‐guided method and the same intron/exon size constraints used by GSNAP (above). The 36 assemblies were merged to a single transcript reference set by Cuffmerge v2.2.1 (Trapnell et al., 2013) with the minimum isoform abundance parameter set to five and further filtered with two criteria: (a) each junction of the transcript is supported by>= 3 reads; (b) The minimum average mapping depth within the alternative region of intron retention events was required to be four if 100% covered, five if 90% covered, and six if 80% covered. The AS event types were classified by AStalavista v3.2 (Foissac & Sammeth, 2007). The percentage of novel junction reads (“complete novel” indicates both the 5’ and 3’ splicing sites are novel, “partial novel” indicates only one splice site is novel) were calculated by RSeQC v2.6.2 (Wang, Wang, & Li, 2012). Differential AS and expression were identified by Cuffdiff v2.2.1 (Trapnell et al., 2013) with default parameters. Supplementary‐dataset1‐differential‐expression‐splicing.xlxs contains the cuffdiff results of all significant differential genes (expression and splicing) for all tests. Heatmaps illustrating the expression of transcription factors and splicing factors were produced using data from cuffdiff results. The ratio of FPKM values from two treatments was used as input for the heatmaps, which were generated using the function heatmap.2 from the gplots R package. Supplementary‐dataset2‐heatmapData contains the data used to generate the heatmaps.

### 2.3 Open reading frame prediction

TransDecoder v3.0.0 (https://transdecoder.github.io/) was used to integrate BLAST and Pfam searches for protein prediction and ORF searches on the filtered transcript assemblies.

### 2.4 Protein interaction network analysis

Genes with significant differential expression and/or AS in response to MeJA treatment were used for protein interaction network construction using STRING v10.0 (Szklarczyk et al., 2015). Genes or their homologs with experimental evidence of protein interaction were connected with lines. Protein interaction networks were displayed using Cytoscape v3.4.0 (Shannon et al., 2003) and annotated with gene expression heatmaps.

### 2.5 miRNA target predication

The collection of 427 *Arabidopsis* miRNAs from the miRNA database (miRBase Release 21, Kozomara & Griffiths‐Jones, 2014) were used as queries to psRNATarget (Dai & Zhao, 2011) to search for potential target sites within the filtered transcript assemblies. Default parameters in scoring schema V2 were used.

### 2.6 Protein extraction, digestion, iTRAQ labeling, and LC‐MS/MS

Proteins were quantified as previously described (Koh et al., 2012), and dissolved in denaturant buffer (0.1% SDS [w/v]) and dissolution buffer (0.5 M triethylammonium bicarbonate, pH 8.5) in the iTRAQ 8‐plex kit (AB sciex Inc.). For each sample, a total of 100 μg of protein were reduced, alkylated, trypsin‐digested, and labeled according to the manufacturer’s instructions (AB Sciex Inc.). The control samples from wild type, *jaz2*, and *jaz7* were labeled with iTRAQ tags 113, 114, and 115, respectively, and the corresponding treated samples were labeled with iTRAQ tags 116, 117, and 118 respectively. In addition, all six samples were mixed and labeled with iTRAQ tag 119 as an internal control. Labeled peptides were desalted with C18‐solid phase extraction and dissolved in strong cation exchange (SCX) solvent A (25% (v/v) acetonitrile, 10 mM ammonium formate, and 0.1% (v/v) formic acid, pH 2.8). The peptides were fractionated using an Agilent HPLC 1260 with a polysulfoethyl A column (2.1 × 100 mm, 5 µm, 300 Å; PolyLC). Peptides were eluted with a linear gradient of 0–20% solvent B (25% (v/v) acetonitrile and 500 mM ammonium formate, pH 6.8) over 50 min followed by ramping up to 100% solvent B in 5 min. The absorbance at 280 nm was monitored and a total of 12 fractions were collected. The fractions were lyophilized and resuspended in LC solvent A (0.1% formic acid in 97% water (v/v), 3% acetonitrile [v/v]). A hybrid quadrupole Orbitrap (Q Exactive Plus) MS system (Thermo Fisher Scientific) was used with high energy collision dissociation (HCD) in each MS and MS/MS cycle. The MS system was interfaced with an automated Easy‐nLC 1000 system (Thermo Fisher Scientific). Each sample fraction was loaded onto an Acclaim Pepmap 100 pre‐column (20 mm × 75 μm; 3 μm‐C18) and separated on a PepMap RSLC analytical column (250 mm × 75 μm; 2 μm‐C18) at a flow rate at 350 nl/min during a linear gradient from solvent A (0.1% formic acid [v/v]) to 30% solvent B (0.1% formic acid (v/v) and 99.9% acetonitrile [v/v]) for 95 min, to 98% solvent B for 15 min, and hold 98% solvent B for additional 30 min. Full MS scans were acquired in the Orbitrap mass analyzer over m/z 400–2000 range with resolution 70,000 at 200 m/z. The top ten most intense peaks with charge state ≥2 were isolated (with 2 m/z isolation window) and fragmented in the high energy collision cell using a normalized collision energy of 28%. The maximum ion injection time for the survey scan and the MS/MS scans were 250 ms, and the ion target values were set to 3e6 and 1e6 respectively. The selected sequenced ions were dynamically excluded for 60 s.

### 2.7 Proteomics data analysis

The raw MS/MS data files were processed by a thorough database searching approach considering biological modification and amino acid substitution against customized *Arabidopsis* database using the ProteinPilot v4.5 with the Fraglet and Taglet searches under ParagonTM algorithm (Shilov et al., 2007). The following parameters were considered for all the searching: fixed modification of methylmethane thiosulfonate‐labeled cysteine, fixed iTRAQ modification of amine groups in the N‐terminus, lysine, and variable iTRAQ modifications of tyrosine. The false discovery rate at the peptide level was estimated with the integrated PSPEP tool in the ProteinPilot Software to be 1.0%. The identified peptide reads were screened for confidence no less than 95%. The screened peptide reads were mapped to TAIR10 primary protein database and peptides which failed to map to the database were regarded as candidates for supporting the AS isoforms. These candidates were manually validated and only reads spanning the AS junction were regarded as evidence for that AS isoform.

## 3 RESULTS

### 3.1 Transcriptomic analysis and genome‐guided assembly

**FIGURE 1:**
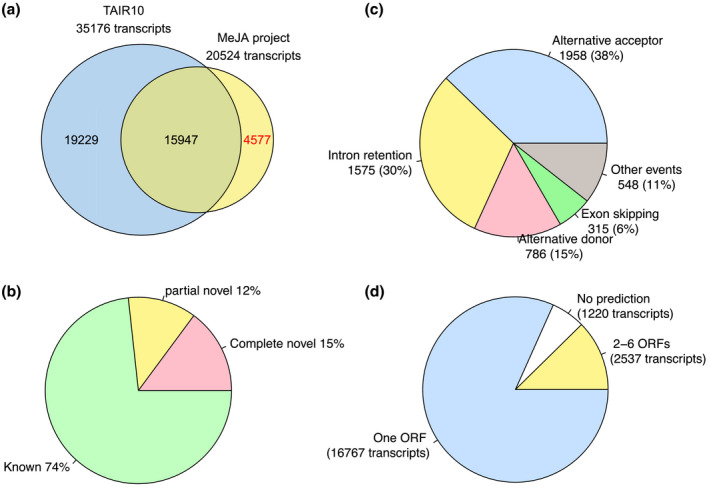
Characterization of assembled transcripts. (a) Comparison of the assembled transcripts from the MeJA RNA‐seq data and TAIR10 annotation. (b) Comparison of the mapped junction reads from the MeJA RNA‐seq data to the TAIR10 annotated junctions. (c) Identified AS patterns detected in the MeJA RNA‐seq assemblies. (d) ORF prediction of the 20,524 transcripts assembled from the MeJA RNA‐seq data with the TransDecoder program (Haas et al., 2013)

452 million reads from 36 RNA‐seq *Arabidopsis* libraries (three each from WT, JAZ2 and JAZ7 roots or shoots w/wo 10 μM methyl jasmonate) were uniquely mapped to the *Arabidopsis* TAIR10 genome assembly (Table S1). These assembled into 20,524 transcripts from 13,647 genes. 15,947 transcripts were previously known (TAIR10) while the remaining 4,577 were novel (Figure 1a). Among all the splice junctions identified in this project, 26% of the junctions have at least one boundary novel to the TAIR10 annotation (Figure 1b). A total of 4446 genes (32.58% of the identified 13,647 genes) demonstrate AS with the majority (98%) of them producing two to five splice variants (Table 1). The most abundant AS events are alternative acceptor (38%) and intron retention (30%; Figure 1c).

**TABLE 1:** Gene isoform number in TAIR10 annotation and in RNA‐seq assembly from this project

Isoform no.	TAIR10		MeJA project	
	Gene no.	Percentage	Gene no.	Percentage
1	21402	78.67	9201	67.42
2	4251	15.63	2998	21.97
3	1133	4.17	881	6.46
4	291	1.07	345	2.53
5	89	0.33	115	0.84
6	26	0.10	65	0.48
7	7	0.03	21	0.15
8	5	0.02	11	0.08
9	1	0.00	6	0.04
10	1	0.00	0	0.00
11	0	0.00	1	0.01
12	0	0.00	2	0.02
13	0	0.00	0	0.00
14	0	0.00	0	0.00
15	0	0.00	1	0.01
Total	27206	100.00	13647	100.00

### 3.2 Jasmonate‐Related Protein Interaction Network

**FIGURE 2:**
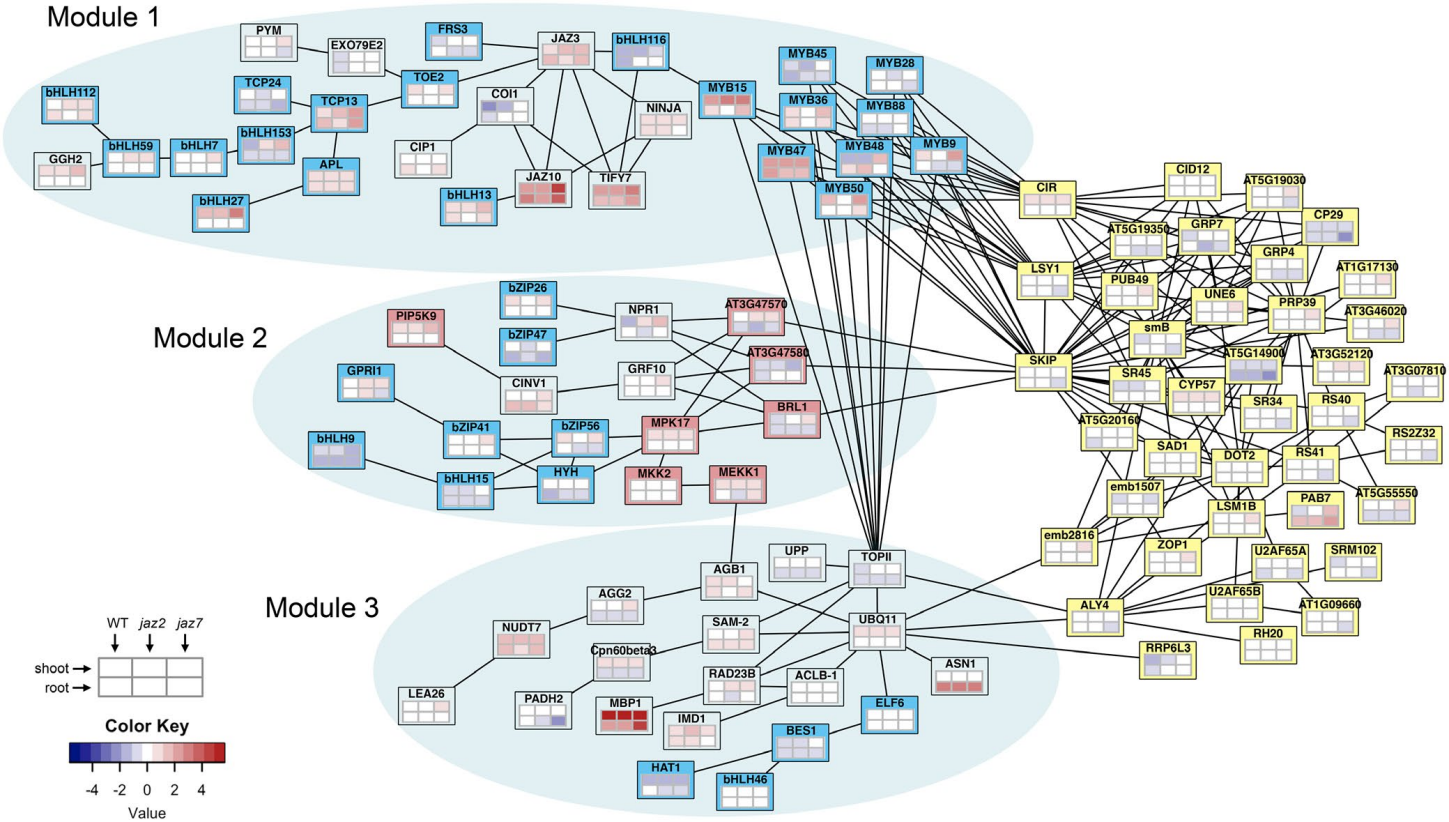
Protein interaction network of genes that undergo AS which show differential expression or differential AS in response to MeJA treatment. Edges indicate experimentally validated interactions of the two connected proteins or their homologs. Yellow boxes indicate proteins related with splicing; blue boxes indicate transcription factors; pink boxes indicate kinase proteins. Heatmaps under the gene name indicate gene expression patterns in response to MeJA treatment

Significant differential gene expression and differential AS were identified with Cuffdiff (Trapnell et al., 2013) in response to MeJA treatment, between tissues, and between genotypes (Table 2). In each case there were always more genes with significant expression changes than genes with significant AS changes. The largest number of differences was observed between tissues. Genes with significant differential expression and/or AS in response to jasmonate treatment were used to generate a protein interaction network with STRING (Szklarczyk et al., 2015). A subset of the network including splicing related proteins, jasmonate key regulatory proteins (JAZs, COI1, and NINJA) as well as genes interacting with them were identified and examined further (Figure 2). We arbitrarily divided the network into four modules. One module contains splicing related genes, and this is connected to three other modules (named Module 1, 2, 3; Figure 2). Module 1 contains the key jasmonate regulatory factors, JAZ3, JAZ10, TIFY7, COI1, and NINJA, as well as transcription factors such as bHLHs and R2R3‐MYBs. Based on the network, the communication between jasmonate signaling and splicing signaling was shown to be mediated by bHLHs, R2R3‐MYBs and three splicing‐related proteins: CBF1‐interacting co‐repressor (CIR), pre‐mRNA‐splicing factor ISY1‐like protein (LSY1) and SNW/SKI‐interacting protein (SKIP). Module 2 mainly contains kinases and transcription factors, and its interaction with the splicing‐related proteins is through kinases and SKIP. Module 3 is centered by a topoisomerase (TOPII) and a ubiquitin (UBQ11) protein. Interaction between the third module and the splicing‐related proteins is through Embryo Defective 2816 (emb2816) and Always Early 4 (ALY4).

**TABLE 2:** Differential AS or expression of genes undergoing AS in treatment comparisons, tissue comparisons and between mutant backgrounds

Genotype	Tissue	Differential alternative splicing				Differential expression
		Splicing diff.	Promoter diff.	CDS diff.	Total	
Untreated vs. 10 μM MeJA						
WT	shoot	44	3	6	48	537
*jaz2*	shoot	32	5	8	37	586
*jaz7*	shoot	234	90	72	326	1400
WT	root	49	25	15	75	886
*jaz2*	root	32	14	9	48	557
*jaz7*	root	43	22	13	67	1131

Genotype	Treatment	Differential alternative splicing				Differential expression
		Splicing diff.	Promoter diff.	CDS diff.	Total	
Shoot vs. root						
WT	No MeJA	159	86	41	249	1943
*jaz2*	No MeJA	162	75	37	243	1896
*jaz7*	No MeJA	246	136	88	389	2049
WT	10 μM MeJA	267	165	84	429	2300
*jaz2*	10 μM MeJA	140	84	53	230	1885
*jaz7*	10 μM MeJA	259	111	68	372	2389

Tissue	Treatment	Differential alternative splicing				Differential expression
		Splicing diff.	Promoter diff.	CDS diff.	Total	
WT vs. *jaz2*, WT vs. *jaz7* or *jaz2* vs. *jaz7*						
Shoot	No MeJA	84	6	25	90	380
Root	No MeJA	37	14	10	53	718
Shoot	10 μM MeJA	172	58	77	231	1096
Root	10 μM MeJA	66	10	22	79	670

### 3.3 Regulation of Transcription Factors (bHLHs and MYBs) and Splicing Factors (SRs and hnRNPs)

**FIGURE 3:**
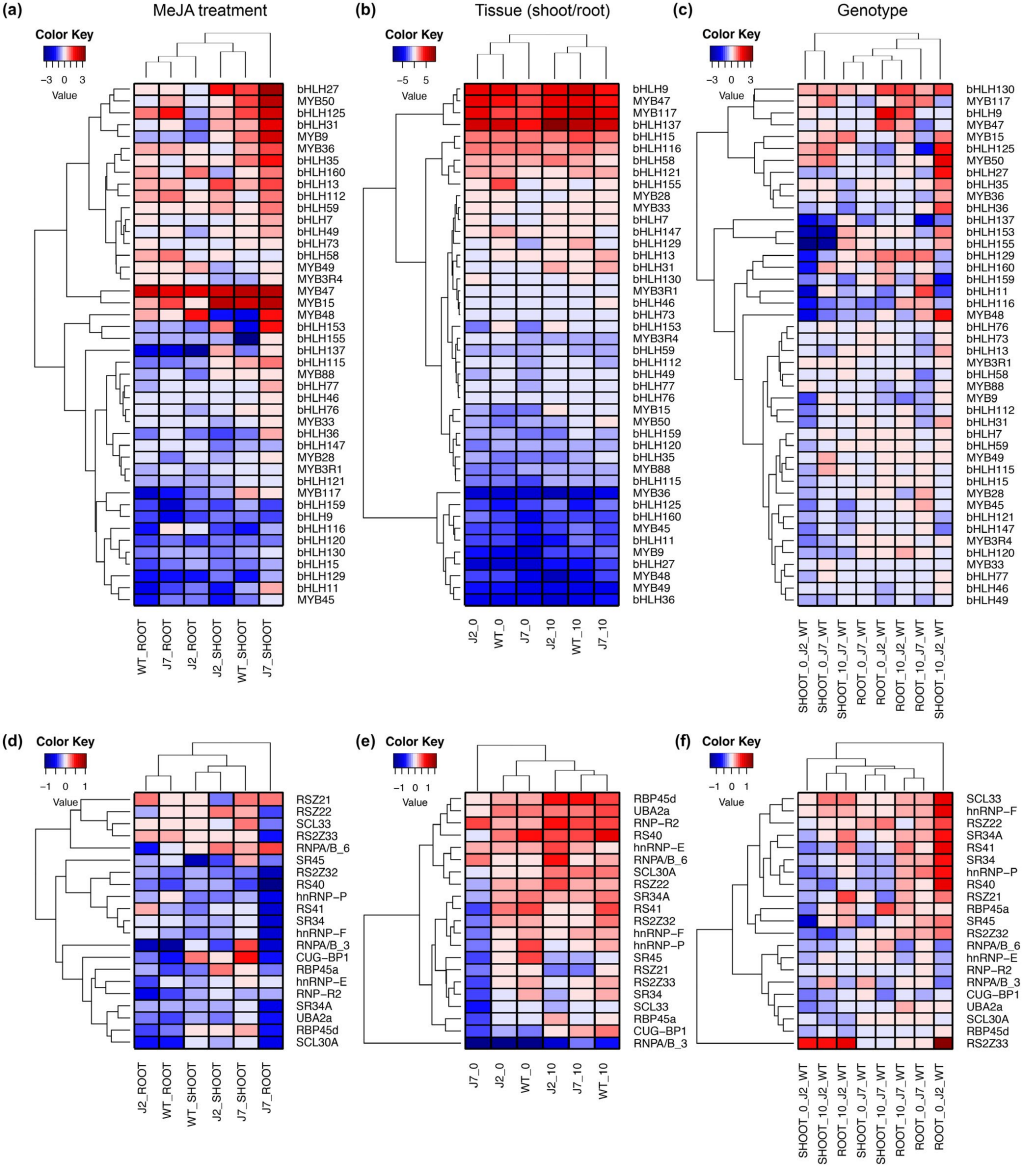
Heatmap of differentially expressed transcription factor (*bHLH* and *MYB*) and splicing factor (*SR* and *hnRNP*) gene family members that undergo AS. Differentially expressed *bHLH* and *MYB* genes under MeJA treatment (a), between tissues (shoot relative to root; b), and between mutant backgrounds (mutant relative to WT; c). Differentially expressed *SR* and *hnRNP* genes under MeJA treatment (d), between shoots and roots; e), and between genotypes (mutants relative to WT; f)

The protein interaction network indicates the inclusion of several members of the bHLH and MYB gene families, suggesting their importance in the jasmonate pathway. Moreover, the SR and hnRNP gene families are key *trans*‐factors involved in the selection of splice junctions and play essential roles in AS regulation (Barbazuk, Fu, McGinnis, 2008). We further analysed the expression patterns of these four important gene families in response to MeJA treatment as well as between tissues and genotypes (Figure 3). Genes without any significant expression changes in any of the three comparisons were not included.

The expression profiles of the MYB and bHLH gene family reveal the impacts of expression in response to MeJA treatment (up or down), as well as whether they act downstream of *JAZ2* and *JAZ7*. For example, while the expression of *bHLH116* is strongly downregulated in WT root, it is slightly upregulated in *jaz2* root, and higher still in *jaz7* root in response to MeJA (Figure 3a). This expression pattern indicates that *bHLH116* might be regulated by *JAZ2* and *JAZ7* in the root and is also supported by the expression change between genotypes (Figure 3c). Similarly, *bHLH77* might be regulated by *JAZ2* and *JAZ7* in the root tissue; *bHLH153*, *bHLH155*, and *bHLH137* might be regulated by *JAZ2* and *JAZ7* in the shoot tissue; *MYB48*, and *bHLH36* are likely regulated by *JAZ7* in both the shoot and root tissues (Figure 3a).

The majority of the splicing factors were downregulated in response to MeJA treatment (Figure 3d). The expression changes were less dramatic in the SR and hnRNP splicing factors compared with the bHLH and MYB transcription factors. However, we observed several splicing factors that may be involved in jasmonate responses: *RBP45a*, *RS2Z33*, *RNPA/B_3*, and *RSZ21*. The expression of *RBP45a* was downregulated in response to MeJA in the WT shoots but exhibits increased expression in the *jaz7* mutant and even higher expression in the *jaz2* mutant. Another interesting case is *RS2Z33*, which is dramatically downregulated in response to MeJA in *jaz7* mutant root tissue but upregulated in the *jaz2* mutant and WT in both shoot and root tissues (Figure 3d). Moreover, the expression of *RS2Z33* in shoot tissue was greatly induced in *jaz2* mutants with respect to WT in the shoot tissue (Figure 3f). The expression of *RNPA/B_3* did not change much in response to MeJA in WT shoot. However, its expression is greatly downregulated in the shoot of *jaz2* and greatly upregulated in the shoot of *jaz7*. These altered expression patterns suggest possible involvement of these splicing factors in the jasmonate pathway regulated by *JAZ2* and *JAZ7*.

### 3.4 Differential alternative splicing in response to MeJA treatment

**FIGURE 4:**
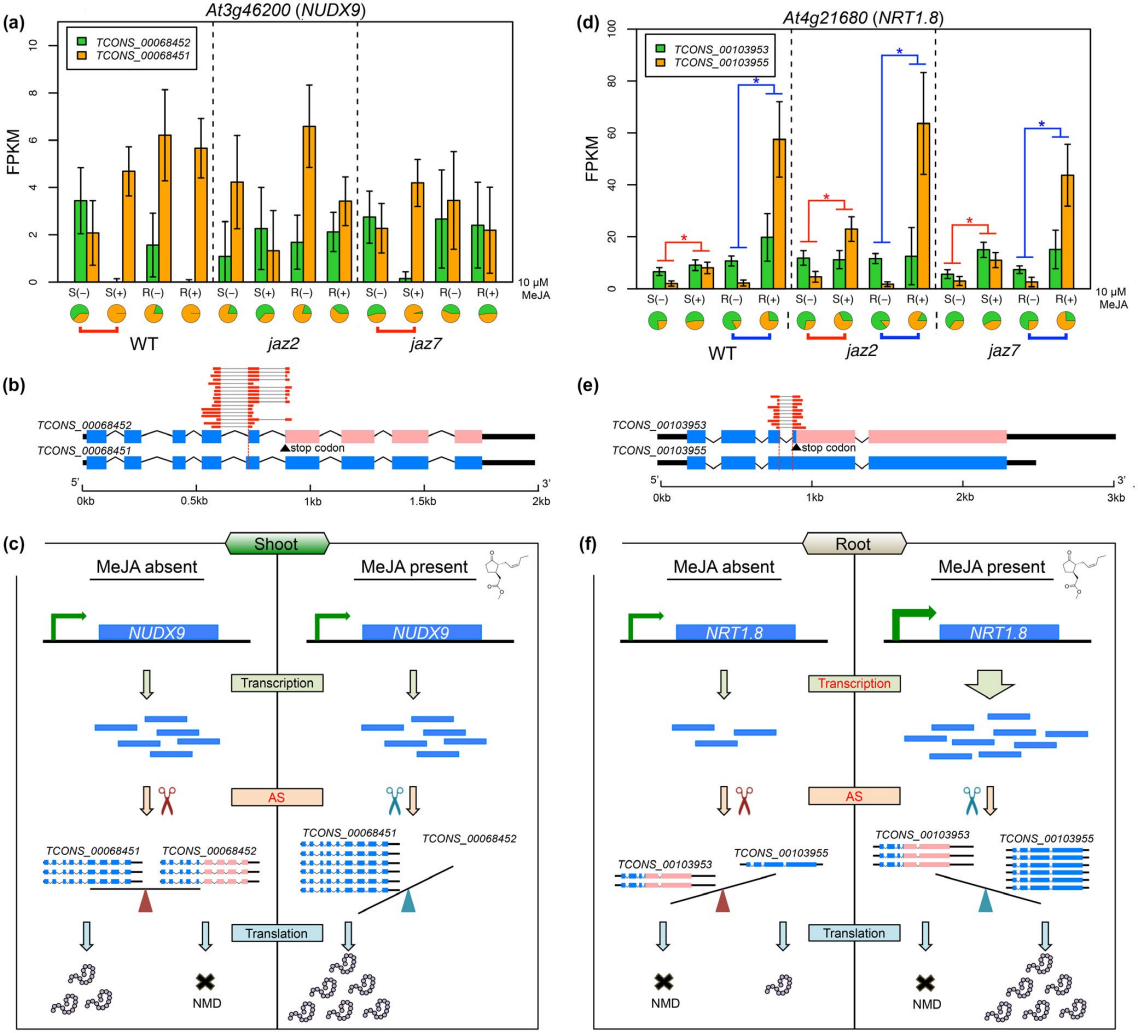
Two genes under AS regulation in response to MeJA treatment. (a) Expression profile of *NUDX9*. Error bars indicate standard deviation. Pie charts under each condition indicate the proportion of each AS isoform relative to the total expression from the locus. Red lines under pie charts indicate significantly differential AS in the shoot tissue. (b) Gene structure of the two isoforms of *NUDX9*. Angled lines indicate introns; thin black boxes indicate UTRs; blue boxes indicate CDS; pink boxes indicate regions which were converted to noncoding regions as a result of an AS induced PTC. Gray line connected red boxes indicate mapped reads supporting the novel junction in the alternative isoform. (c) Regulation of *NUDX9* in response to MeJA treatment by AS. (d) Expression profile of *NRT1.8*. Blue lines under pie charts indicate significantly differential AS patterns in the root tissue. Red and blue lines above the barplot indicate significantly differential gene expression in shoot and root respectively. (e) Gene structure of the two isoforms of *NRT1.8*. (f) Regulation of *NRT1.8* in response to MeJA treatment by transcription and AS

The highest number of significantly differential AS events in response to MeJA treatment was observed in shoot from *jaz7* mutants (Table 2). Across each of the six treatments (two tissues sampled from each of three genotypes), only a fraction of the differential AS events are shared in response to MeJA (Figure S1). Genes with significant differential AS in response to MeJA in more than one genotype of a specific tissue (shoot or root) were analyzed further (Figure S2). Among the 16 genes, seven genes have AS events in the coding region, while five have events that could lead to a PTC that could target these transcripts for NMD (Kervestin & Jacobson, 2012). Based on the AS pattern and expression profiles, we observed cases where AS regulation alone may regulate gene expression (through NMD), such as *NUDIX HYDROLASE HOMOLOG 9* (*NUDX9*; Figure 4a), and cases where AS and transcriptional regulation together regulate gene expression, such as *NITRATE TRANSPORTER 1.8* (*NRT1.8*; Figure 4d).

Two splice variants were identified for gene *NUDX9* with an alternative acceptor event in the fifth exon leading to a frame shift that results in a PTC in one of the AS transcripts. There are 17 mapped reads supporting the AS junction (Figure 4b). While no significant changes in the gene expression level were identified in response to MeJA, we observed significant differential AS in the shoot tissue of WT and *jaz7* upon MeJA treatment (Figure 4a). In shoot of WT and *jaz7*, When there is no MeJA treatment the two splice variants are present at similar levels, with one isoform (the primary isoform annotated by TAIR10) generating a protein product, while the alternative acceptor AS isoform is potentially subject to NMD. However, upon MeJA treatment almost all isoforms are the primary isoform providing the potential to generate twice as much productive translation of the protein product under MeJA treatment relative to untreated tissue with no required increase in the rate of transcription (Figure 4c).

*NRT1.8* exhibited both differential expression and AS in response to MeJA treatment in the root of the three genotypes (Figure 4d). Two splice variants were identified for *NRT1.8*. The AS isoform contains an intron retention and an alternative polyadenylation event compared with the primary annotated isoform. The removal of the retained intron in the AS isoform, which is supported by 10 mapped reads (Figure 4e), leads to PTC and thus the AS isoform is unproductive. Upon MeJA treatment, the expression level of *NRT1.8* was up‐regulated and AS generated more of the productive isoform. In the case of *NRT1.8*, differential transcription and AS both play a role to potentially generate more protein products in response to increased jasmonate (Figure 4f).

### 3.5 Alternative splicing variants differentially targeted by miRNA

We used psRNATarget (Dai & Zhao, 2011) to predict miRNA targets on the filtered transcript assemblies. A total of 508 genes were predicted to be targeted by miRNAs, among which 171 have evidence of AS (Figure S3). Sixty‐four genes contain miRNA target sites subjected to AS regulation, suggesting the potential of being regulated by a combination of AS and miRNA. Among them, 21 showed significantly changed isoform proportions (significantly differential AS identified by Cuffdiff) in MeJA treatment/tissue/genotype comparisons (Figure S4). Most of the changes in isoform ratio occur between tissues, with only five genes—*SCHLAFMUTZE* (*SMZ*), *ALDEHYDE OXIDASE 2* (*AAO2*), *PLANT U‐BOX 4* (*PUB4*), *LON PROTEASE 2* (*LON2*), *At3g02740*—exhibiting differences in relation to MeJA treatment and three (*AAO2*, *LON2,* and *CEST*) exhibiting differences between genotypes (Figure S4).

### 3.6 Alternative splicing variants with novel functions

In order to identify AS isoforms with novel functions, the ORF and protein products of the 20,524 transcripts were predicted with TransDecoder (Figure 1d; https://transdecoder.github.io/). For transcripts shared with TAIR10 database, 99.6% predicted products from TransDecoder match the TAIR10 annotation, supporting the efficacy of TransDecoder. We used the ORF annotation from TAIR10 for the 15,947 known transcripts and the ORF prediction from TransDecoder for the remaining 4,577 novel transcripts. Transcripts with no ORF prediction or multiple ORF predictions were excluded from further analysis, thus 3,220 genes with evidence of undergoing AS (a total of 7,638 transcripts) with ORF predictions from either TAIR10 or TransDecoder were used for further analysis.

Not all AS leads to multiple protein products of a gene. For example, AS events that affect UTR regions will not generate a novel protein, and those that result in a PTC leading to NMD are not translated. In this study, 37.6% of the AS events occurred in UTRs (Figure S5). Long 3’‐UTRs and introns in the UTR region are *cis*‐elements that trigger NMD (Kalyna et al., 2012; Kervestin & Jacobson, 2012). Two experimentally validated criteria for NMD in *Arabidopsis* were applied to identify potential cases of NMD (Kalyna et al., 2012): (a) 3’ UTR length >350 nt; and (b) the distance of stop codon to last exon junction >55 nt (Figure S5). As a result, a total of 1,745 transcripts from 1,106 genes (34.3% genes with evidence of AS) were predicted to be targets of NMD. After eliminating genes undergoing AS but producing only a single protein product, there were 1,464 genes with AS transcripts that can potentially generate multiple protein products.

**FIGURE 5:**
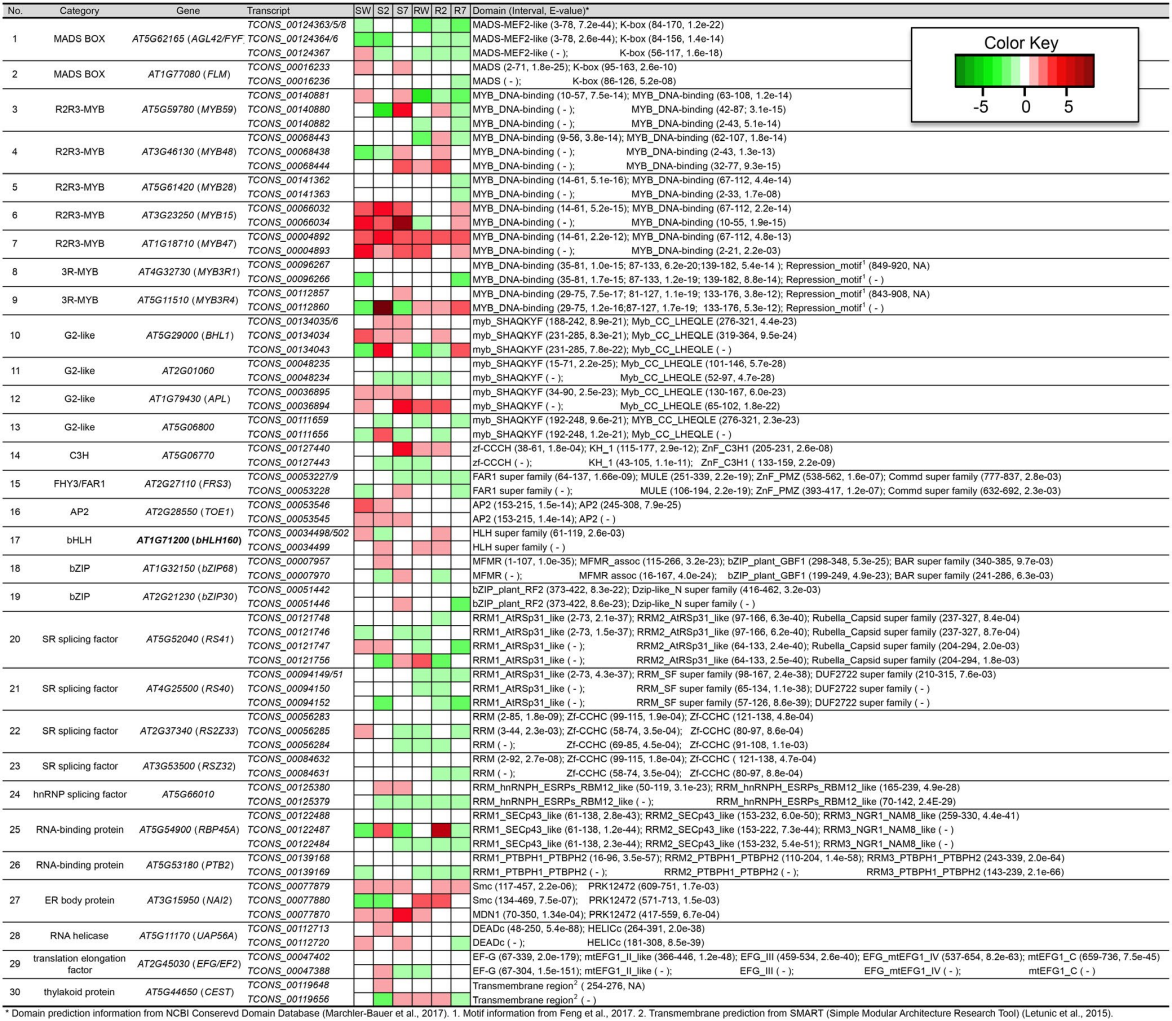
AS Genes with different domain structures predicted between transcript isoforms. Heatmaps indicate up‐ or down‐regulated transcript expression in response to MeJA treatment in six conditions (SW: shoot of WT; S2: shoot of *jaz2*; S7: shoot of *jaz7*; RW: root of WT; R2: root of *jaz2*; R7: root of *jaz7*)

Among these 1,464 genes, 171 were identified to be splicing‐related genes, jasmonate‐related genes, or transcription factors, and also show significant differential expression and/or AS in response to jasmonate treatment/tissues/genotypes comparison. Predicted proteins from the 372 transcripts of these 171 genes were analyzed with the NCBI Conserved Domain Database (CDD, Marchler‐Bauer et al., 2015) and Simple Modular Architecture Research Tool (SMART, Letunic, Doerks, & Bork, 2015) to determine their domain structure. We identified potential novel AS isoforms by two criteria: (a) the AS protein has conserved domain(s) which suggests functional importance, and (b) the domain arrangement of the AS variant is different from the primary protein, indicating functional divergence. A total of 30 genes were identified that satisfy both criteria (Figure 5). We observed conservation between members of the same gene family for domain pattern changes. For example, the MADS‐box genes *FOREVER YOUNG FLOWER* (*FYF*) and *FLOWERING LOCUS M* (*FLM*) both have AS isoforms lacking the MADS domain; R2R3‐MYB genes *MYB59*, *MYB48*, *MYB28*, *MYB15*, and *MYB47* all have AS isoforms lacking the R2 domain; 3R‐MYB genes *MYB3R1* and *MYB3R4* both have AS isoforms lacking the C‐terminal repression motif (Figure 5).

### 3.7 Alternative splicing variant of bHLH160 with a potential novel function

**FIGURE 6:**
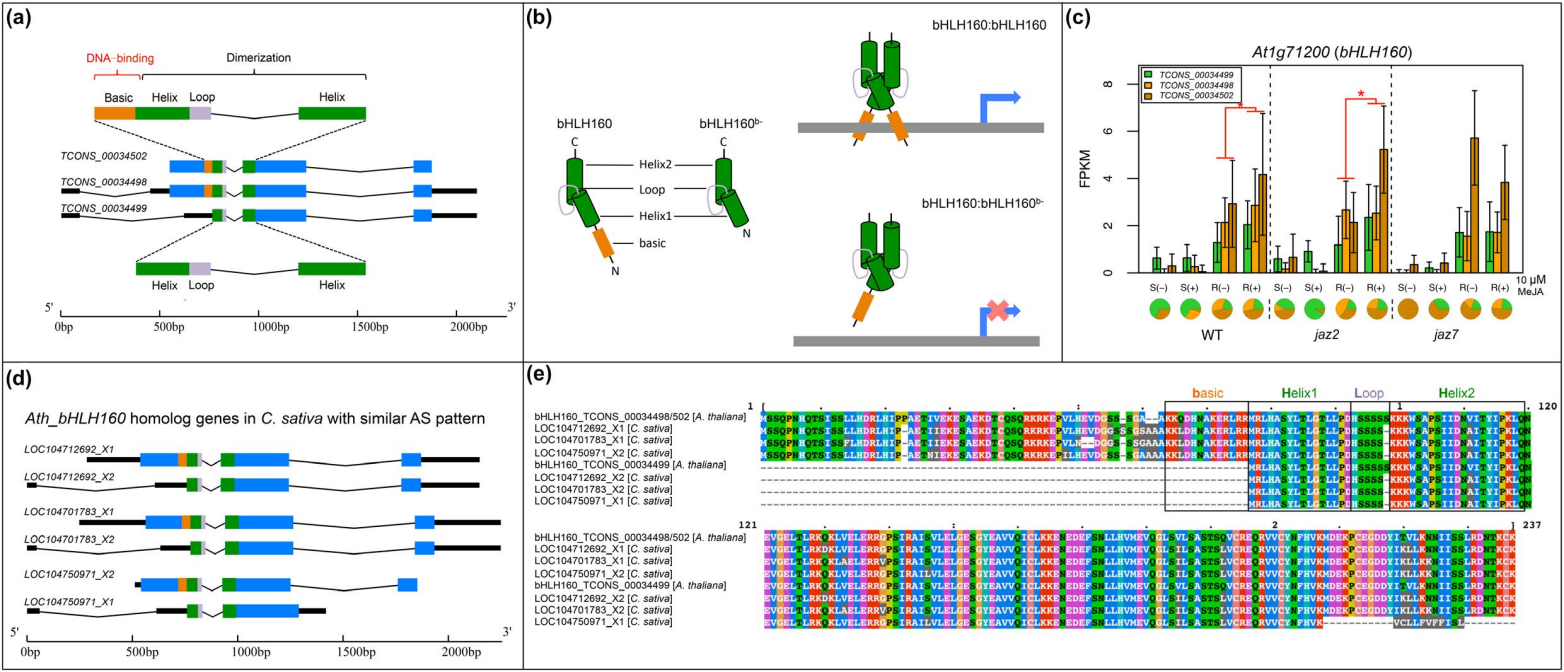
*Arabidopsis bHLH160* AS patterns and the proposed regulatory function of the splice variant. (a) Gene structure of the transcript isoforms of *Arabidopsis bHLH160*. (b) Protein 3D structure of the *Arabidopsis* bHLH160 proteins adapted from Ma et al., 1994 and their regulatory roles. (c) Expression profile of *Arabidopsis bHLH160*. Error bars indicate the standard deviation. Pies charts under each condition indicate the proportion of each AS isoform present relative to the total expression from the locus. Red lines above the barplot indicate significantly changed gene expression. (d) Gene structure of the transcript isoforms of *Camelina sativa LOC104712692*, *LOC104701783,* and *LOC104750970*. Angled lines indicate introns; black thin boxes indicate UTRs; thick boxes indicate CDS; orange, green, and purple indicate basic, helix, and loop, respectively, in the bHLH domain. (e) Multiple sequence alignments of the protein isoforms from *Arabidopsis thaliana* bHLH160, *Camelina sativa* LOC104712692, LOC104701783, and LOC104750970. The bHLH domain is indicated within black boxes

Among the 30 genes that were predicted to produce AS isoform(s) with novel functions, we identified *bHLH160* as an interesting case. Like all bHLH proteins, the bHLH160 contains a basic‐Helix‐Loop‐Helix domain, where the basic region is responsible for DNA binding, and the HLH region is responsible for dimerization (Figure 6a, Carretero‐Paulet et al., 2010). When the bHLH dimer stably binds with the DNA recognition sequence, it serves as a transcriptional activator/repressor. Three transcript isoforms of *bHLH160* were assembled with two of them (*TCONS_00034502* and *TCONS_00034498*) predicted to generate the primary protein product and the third isoform (*TCONS_00034499*) predicted to produce a product lacking part of the N‐terminal region, including 13bp basic region and its upstream sequences (Figure 6a). There are 45 mapped reads supporting the AS junction of the third isoform (Figure S6). Given the function of the basic and HLH regions, a bHLH protein that lacks the basic region is able to dimerize but unable to bind DNA. In addition, by dimerizing with normal bHLH proteins, bHLH160^b‐^ is expected to decrease the active bHLH dimers (Figure 6b). Thus bHLH160^b‐^ functions in a manner opposite to that of the primary isoform. We observed significantly upregulated gene expression of *bHLH160* in response to MeJA treatment in WT and *jaz2* mutant roots (Figure 6c).

To investigate whether the AS regulatory pattern in bHLH160 is conserved among other bHLH genes, all other bHLH genes expressed in these data with evidence of AS were examined and no similar AS pattern was observed. Phylogenetic analysis of the bHLH gene family indicates *bHLH160* is a newly evolved gene in Brassicaceae (Carretero‐Paulet et al., 2010). We further checked the *bHLH160* orthologs in other Brassicaceae species and three genes with similar AS patterns based on the gene annotation (NCBI database) of *Camelina sativa* were identified (Figure 6d). Protein sequence alignments of the primary and AS isoforms of the *Arabidopsis bHLH160* and the three *Camelina* genes (*LOC104712692*, *LOC104701783*, *LOC104750971*) suggest a conservative alternative start codon at the first amino acid of Helix1 (Figure 6e). A similar AS pattern observed in multiple species suggests that AS generation of both an activator and a repressor might be a conserved and important regulatory mechanism in *bHLH160* and its orthologs.

### 3.8 Proteomics validation of alternatively spliced isoforms

**FIGURE 7:**
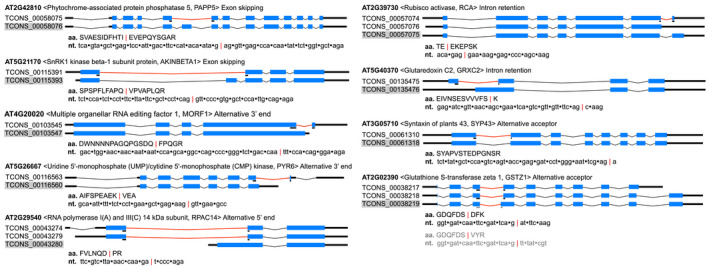
Proteomics validated AS isoform expression. Gene structure was displayed by Gene Structure Display Sever 2.0 (http://gsds.cbi.pku.edu.cn, Hu et al., 2013): thick black line—UTR; blue box—exon; angled line—intron; red angled line—intron splitting the junction of the transcript isoform supported by proteomics data; black/gray line under blue box—peptide mapped region. Shaded isoform name indicates primary annotated isoform from TAIR10 annotation

An attempt was made to validate AS events identified at the transcript level with proteomics data. We applied three criteria to select peptide sequences supporting identified AS events: (a) The peptide sequence should have at least a 95% confidence level; (b) The peptide sequence is uniquely mapped to a single gene; (c) The peptide sequence is mapped to the AS junction of the AS isoform. With the above criteria, AS events of nine genes were identified to have evidence supporting the protein level expression (Figure 7). Of the nine AS events, two (*PAPP5*, *AKINBETA1*) are exon skipping; two (*RCA*, *GRXC2*) are intron retention; two (*SYP43*, *GSTZ1*) are alternative acceptor, two (*MORF1*, *PYR6*) are alternative polyadenylation, and one (*RPAC14*) is alternative promoter (Figure 7). Among them, *GSTZ1* has support for both the AS junction and the primary annotated splicing junction. Two (*MORF1* and *RPAC14*) of the nine genes showed differential AS in response to MeJA and/or genotype comparison (Figure S7).

## 4 DISCUSSION

### 4.1 Alternative splicing profiles and networks

**FIGURE 8:**
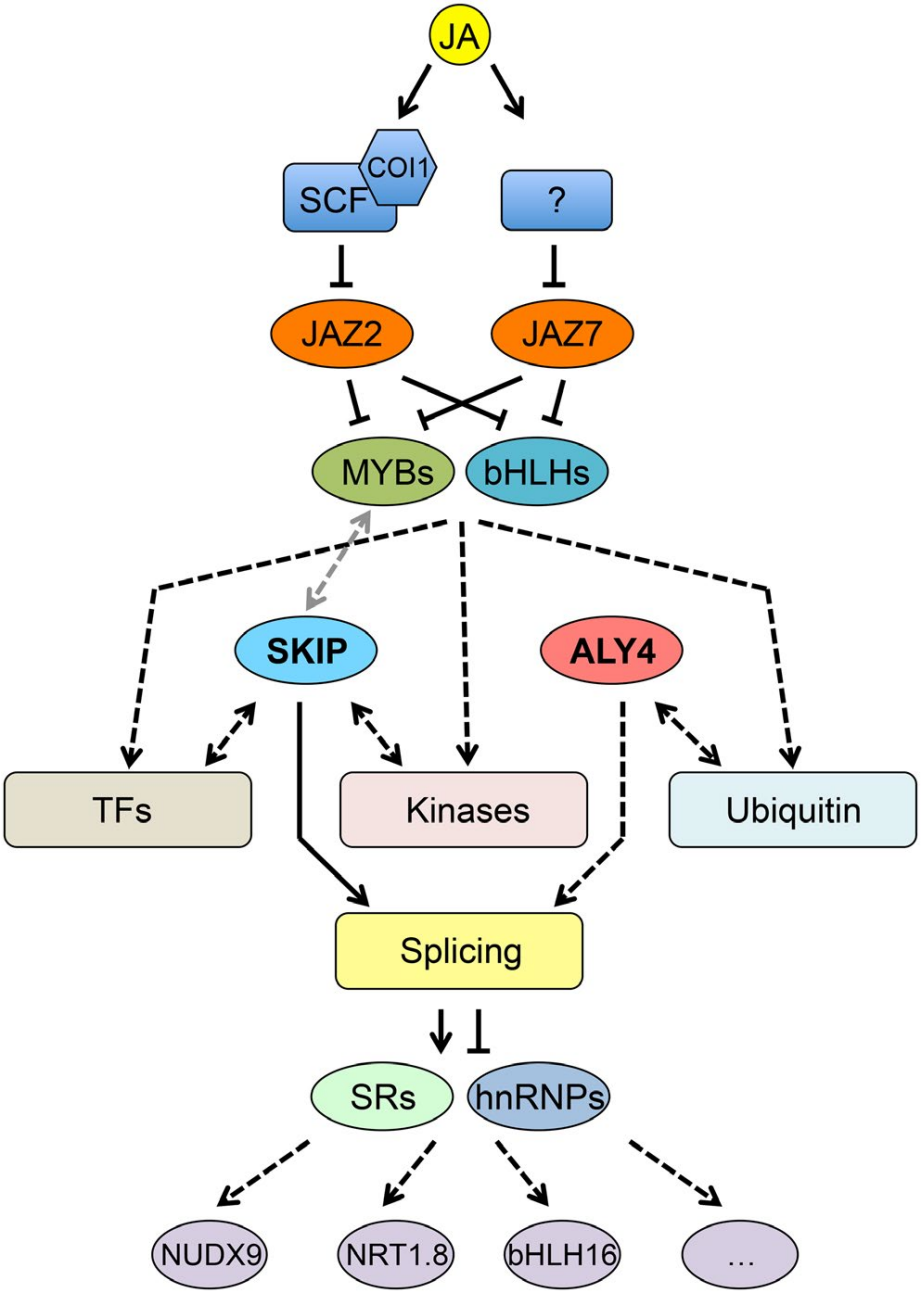
Model of alternative splicing regulation in the jasmonate signaling pathway mediated by JAZ2 and JAZ7. JA‐Ile (JA) negatively regulates JAZ2 by SCF COI1 complex and JAZ7 by other unknown pathways. JAZ2/7 represses the expression of transcription factor MYBs and bHLHs. These two types of transcription factors further regulate downstream pathways mediated by transcription factors, kinases, or ubiquitin. SKIP could interact with the transcription factor and kinases mediated pathways and ALY4 could interact with the ubiquitin mediated pathways. The SKIP and ALY4 are directly or indirectly involved in splicing regulation, which regulates alternative splicing of targeted genes (e.g. NUDX9, NRT1.8—Figure 4; bHLH16—Figure 6) by splicing factors SR or hnRNP family proteins

Focusing on jasmonate signaling core regulators and splicing related genes, a protein network was identified (Figure 2). The bHLH and MYB transcription factors likely mediate transmission of the jasmonate signal to the splicing‐related genes through splicing factors CIR (At2g44200), LSY1 (At3g18790), and SKIP (Ag1g77180; Figure 2). SKIP is an SNW domain‐containing protein, which has conserved functions as both a transcriptional co‐regulator (Li et al., 2016; Lim et al., 2009) and a splicing factor (Cui et al., 2017; Feng et al., 2015; Li et al., 2016; Wang, Wu, et al., 2012). In *Arabidopsis*, SKIP is a key regulator of AS across a broad collection of regulated processes, such as abiotic stress response (Feng et al., 2015; Lim et al., 2009), circadian clock (Wang, Wu, et al., 2012), light signaling (Zhang et al., 2014), and flowering time (Cui et al., 2017). SKIP is a spliceosome component that physically interacts with SR family splicing factors, such as SR45 (Wang, Wu, et al., 2012), and a variety of its target pre‐mRNAs (Cui et al., 2017; Feng et al., 2015; Wang, Wu, et al., 2012). SKIP regulates AS by controlling recognition and cleavage of the 3’ and 5’ splicing sites (Feng et al., 2015) and the *skip‐1* mutant in *Arabidopsis* causes genome‐wide splicing defects (Wang, Wu, et al., 2012). In the regulatory network (Figure 2), SKIP is a hub protein connecting with the most proteins including splicing‐related proteins, transcription factors (module 1), and kinases (module 2). Considering its function and placement in the network, perhaps SKIP may serve as a key regulator mediating jasmonate signaling through alternative splicing regulation (Figure 8). The third module is centered by a polyubiquitin UBQ11 (At4g05050) and a topoisomerase TOPII (At3g23890), which are connected to the splicing‐related proteins mainly through two genes: emb2816 (At2g03870) and ALY4 (At5g37720; Figure 2). *emb2816* is a SM‐like gene that encodes a component for the U6 snRNP (Perez‐Santángelo et al., 2014). Homologs of emb2816 in *Arabidopsis* and the human ortholog of emb2816 regulate circadian rhythms by regulating RNA splicing (Perez‐Santángelo et al., 2014). Jasmonate is an important phytohormone involved in plant circadian regulation that demonstrates peak expression at midday in preparation for potential insect feeding, which is most probable at that time (Lu, McChung, & Zhang, 2017). The presence of emb2816 in the protein network may be indicative of a possible regulatory pathway for jasmonate signaling in circadian rhythm regulation although further experiment validation is needed. ALY4 is a key regulator involved in nucleo‐cytosolic mRNA transport (Pfaff et al., 2018) and transcriptional regulation (Storozhenko, Inzé, Montagu, & Kushnir, 2001). In *Arabidopsis*, mutation in *ALY* genes leads to multiple vegetative and reproductive defects (Pfaff et al., 2018). Based on its function, ALY4 could potentially regulate TOPII and UBQ11 through transcriptional regulation and/or mRNA transportation (Figure 2). It is interesting that SKIP and ALY4, which are key regulators connecting splicing signaling with other genes, have both RNA‐binding and DNA‐binding affinity. The broad binding affinity may confer these genes with greater impact on the gene cascade by serving as a hub which can integrate multiple signals.

Taken together, the three modules interacting with the splicing‐related proteins suggest three possible signal transduction pathways between jasmonate and mRNA splicing regulation: transcription factors (module 1), kinases (module 2), and ubiquitin pathways (module 3; Figures 2 and 8). The first two modules are likely mediated by a key regulatory SKIP and the third module by another key regulator ALY4. Further functional validation of the two proteins will be important for our understanding of AS in the jasmonate signaling pathway.

### 4.2 Alternative splicing coupled miRNA regulation

In this study, we identified 64 genes which contain miRNA binding sites potentially regulated by AS, including the known *SPL4* (Figure S8; Wu & Poethig, 2006). In *Arabidopsis*, alternative polyadenylation on the 3’UTR of *SPL3/4/5* affects the *miR156* binding site and generates miR156‐sensitive and miR156‐insensitive isoforms. Increased abundance of miR156‐insensitive isoforms causes juvenile‐to‐adult transition (Wu & Poethig, 2006). Although jasmonate is known to be an important negative regulator in juvenile‐to‐adult transition (Hibara et al., 2016), we did not observe a significantly changed level of the miR156‐insensitive form of *SPL4* (*TCONS_00010799*) in response to jasmonate treatment (Figure S8). Whether AS‐coupled miRNA regulation in *SPL4* is regulated by jasmonate remains to be explored.

We identified a small proportion of genes (32 genes, 6.3% of miRNA regulated genes) that were predicted to contain multiple miRNA target sites. Most plant miRNA regulated genes were regulated by only one miRNA with a single target site (Axtell, Westholm, & Lai, 2011; Voinnet, 2009). Plant genes containing multiple miRNA binding sites were rarely reported (Campo et al., 2013). In rice, the dominant splicing variant of *Nramp6* (*Natural resistance‐associated macrophage protein 6*, *Os01g31870*), *Nramp6.8*, contains two *miR7695* binding sites in its 3’UTR region. The expression level of *Nramp6.8* is negatively correlated with *miR7695*, and the over‐expression of *miR7695* confers pathogen resistance in rice (Campo et al., 2013). Gene *AAO2* identified in this work showed significantly changed isoform proportion in response to MeJA treatment in the shoot of *jaz7*, and it was predicted to contain two miRNA target sites recognized by different miRNAs, *miR414* and *miR5021* (Figure S3). The miRNA binding sites are predicted to be on the 3’UTR of one of the two transcript isoforms. AAO2 is one of the four *Arabidopsis* aldehyde oxidase genes (AAO1, AAO2, AAO3, and AAO4) potentially involved in the last step of biosynthesis of indole‐3‐acetic acid (IAA) and/or abscisic acid (ABA; Akaba et al., 1999; Seo et al., 2004). One possible explanation is that AAO2 is under AS and miRNA regulation and may be involved in the interactions among jasmonate, IAA, and ABA signals.

In animals, AS‐associated miRNA regulation is known to play important roles in a wide variety of pathways, such as immune cell activation, cell proliferation, muscle stem cell function, and heart development (Boutet et al., 2012; Kalsotra, Wang, Li, & Cooper, 2010; Sandberg, Neilson, Sarma, Sharp, & Burge, 2008). In *Arabidopsis*, more than 12.4% of miRNA binding sites are predicted to be regulated by AS (Yang, Zhang, & Li, 2012). However, evidence identified for AS‐coupled miRNA regulation in response to MeJA treatment in this study is limited (Figure S4). Moreover, many of the predicted miRNA target sites were located at the long 3’UTRs of the AS isoforms (e.g. AAO2, GRF3, STR16, Figure S3). These long 3’UTRs themselves can trigger NMD (Kalyna et al., 2012). It is possible that miRNAs may be important regulators, working alongside the NMD pathway, to reduce the abundance of transcripts with long 3’UTRs. It has been shown that miRNAs target introns in *Arabidopsis* and rice (Meng, Shao, Ma, & Wang, 2013). Taken together, these results suggest that miRNA may specifically repress or degrade erroneous mRNA with intron or long 3’UTR generated by AS.

### 4.3 Functional AS regulation

Several cases were identified where AS may play functional roles in response to MeJA. In terms of AS regulation—determined by a changed proportion of splice variants under different conditions—we identified genes *NUDX9* and *NRT1.8* (Figure 4). Both genes exhibited significantly altered isoform proportions upon increased jasmonate, suggesting existence of *cis*‐elements (intronic/exonic splicing enhancer/silencer) and different involvement of *trans*‐factors (e.g. SR splicing activators and hnRNPs splicing repressors). Specifically, we observed significantly changed expression of SR genes *RS2Z33*, *RSZ21*, and hnRNP genes *RBP45a*, *RNPA/B_3* in response to MeJA treatment (Figure 3). These splicing factors are candidate genes responsible for differential AS induced by jasmonate. Moreover, we explored a protein interaction network of jasmonate‐responsive genes (Figure 2). In this scenario, increased jasmonate would trigger the jasmonate signaling pathway. The signal could then be mediated and transferred via transcription factors, kinases and ubiquitin signaling pathways and ultimately transmitted to splicing‐related factors that would regulate AS of the responsive genes, such as *NUDX9* and *NRT1.8* (Figures 4 and 8). Interestingly, the AS isoforms of both genes are potentially subjected to NMD due to a PTC. NMD‐coupled AS is an efficient mechanism to downregulate gene expression (Kalyna et al., 2012; Kervestin & Jacobson, 2012) and it was predicted to occur in 34.3% of the genes demonstrating AS in this analysis (Figure S5). These AS isoforms would escape proteomics validation due to lack of protein products. NRT1.8 is a nitrate transporter which enhances nitrate uptake by mediating nitrate unloading from the xylem vessels (Li et al., 2010). Specifically, *NRT1.8* not only shows differential AS but also increased transcription in response to MeJA treatment. The two regulation points (transcription and AS) function synergistically to increase NRT1.8 protein product (Figure 4). Increased NRT1.8 protein level indicates its possible involvement in jasmonate‐triggered plant nitrate uptake. NUDX9 is a GDP‐_D_‐Man pyrophosphohydrolase, which indirectly modulates ammonium responses by hydrolysis of GDP‐_D_‐Man in the root (Tanaka et al., 2015). Here, we reported a possible involvement of *NUDX9* in the jasmonate signaling pathway by AS regulation in the shoots of *Arabidopsis*.

In addition to regulating the relative abundance of isoforms, AS also plays important roles by generating functionally novel splice variants, as is the case for the well documented JAZ repressors (Chung et al., 2010; Chung & Howe, 2009; Moreno et al., 2013; Yan et al., 2007). Notably, in response to wounding, the ratio of the expressed *JAZ10* splice variants did not change significantly (Yan et al., 2007). The AS regulation of the JAZ repressors lies in generating a stable repressor (lacking the domain recognized by COI1) in addition to the primary degradable repressor rather than modulating the proportion of isoforms. AS has the potential to generate isoforms with different functions by modulating domain structures (S. R. Thatcher, Danilevskaya, et al., 2016). By identifying AS genes with different domain arrangements between splice variants, an attempt was made to identify cases similar to JAZ repressors in the jasmonate signaling pathway. We identified a copper‐responsive transcription factor *bHLH160* (Bernal et al., 2012; Yamasaki, Hayashi, Fukazawa, Kobayashi, & Shikanai, 2009), which has the potential to generate an activator and a repressor through AS by inclusion/exclusion of sequences coding for the DNA‐binding domain (Figure 6). The splice variant *bHLH160^b‐^* contains the dimerization domain but lacks the DNA‐binding domain, thus it would repress the primary isoform function by dimerizing with primary *bHLH160* transcripts but not able to bind with their *cis*‐regulatory elements. Human *Id* genes, which only contain the HLH domain like *Arabidopsis bHLH160^b‐^*, function in a similar manner to repress gene expression (Sikder, Devlin, Dunlap, Ryu, & Alani, 2003). Interestingly, similar AS regulation was observed in MADS‐box genes, *FYF* and *FLM*. The AS isoforms of *FYF* and *FLM* have lost the MADS‐box domain, which is the DNA‐binding domain, but retained the K‐box domain, which facilitates dimerization of MADS‐box proteins (Par̆enicová et al., 2003; Figure 5). Thus, the splice variants of *FYF* and *FLM* may negatively regulate the original function of the gene in a manner similar to *bHLH160^b‐^*. AS could also result in isoforms that have the same outcome but different strengths, which is the case for *MYB3R1* and *MYB3R4* (Figure 5). *MYB3R1* and *MYB3R4* are transcriptional activators which upregulate G2/M transition in cell cycle (Haga et al., 2007). AS of *MYB3R1* and *MYB3R4* leads to truncated proteins lacking the repression motif, which would serve as hyper‐activators compared with the primary proteins (Feng, Burleigh, Braun, Mei, & Barbazuk, 2017; Kato et al., 2009). In most of the identified cases, the primary protein product of the genes has multiple domains and the AS isoform has lost one or more domain(s; Figure 5). It is not surprising that many of the cases we identified are transcription factors and splicing factors, as they usually contain at least two domains: the DNA/RNA‐binding domain and the regulatory domain. Based on these observations, AS could serve as an important regulator on multi‐domain proteins that could generate splice variants with different functions by altering domain arrangement. Further functional validation of these cases would help to elucidate regulatory roles of different isoforms and their involvement in the jasmonate signaling pathway.

## AUTHOR CONTRIBUTIONS

GF, SC, WBB designed the work. GF, MJY, JK, RD generated data. QF, MJY, JK, RD, LB, SC, WBB analyzed and reviewed the data. GF, SC, and WBB wrote the manuscript.

## Supporting information

Supplementary Material

Supplementary Material

Supplementary Material

## References

[pld3245-bib-0001] Akaba, S. , Seo, M. , Dohmae, N. , Takio, K. , Sekimoto, H. , Kamiya, Y. , … Koshiba, T. (1999). Production of homo‐ and hetero‐dimeric isozymes from two aldehyde oxidase genes of *Arabidopsis thaliana* . Journal of Biochemistry, 126, 395–401. 10423535 10.1093/oxfordjournals.jbchem.a022463

[pld3245-bib-0002] Attaran, E. , Major, I. T. , Cruz, J. A. , Rosa, B. A. , Koo, A. J. K. , Chen, J. , … Howe, G. A. (2014). Temporal dynamics of growth and photosynthesis suppression in response to jasmonate signaling. Plant Physiology, 165, 1302–1314. 24820026 10.1104/pp.114.239004 PMC4081338

[pld3245-bib-0003] Axtell, M. J. , Westholm, J. O. , & Lai, E. C. (2011). Vive la différence: biogenesis and evolution of microRNAs in plants and animals. Genome Biology, 12, 221. 21554756 10.1186/gb-2011-12-4-221 PMC3218855

[pld3245-bib-0004] Bai, Y. , Meng, Y. , Huang, D. , Qi, Y. , & Chen, M. (2011). Origin and evolutionary analysis of the plant‐specific TIFY transcription factor family. Genomics, 98, 128–136. 21616136 10.1016/j.ygeno.2011.05.002

[pld3245-bib-0700] Barbazuk, W. B. , Fu, Y. , & McGinnis, K. M. (2008). Genome‐wide analyses of alternative splicing in plants: Opportunities and challenges. Genome Research, 18, 1381–1392. 18669480 10.1101/gr.053678.106

[pld3245-bib-0005] Bernal, M. , Casero, D. , Singh, V. , Wilson, G. T. , Grande, A. , Yang, H. , … Connolly, E. L. et al (2012). Transcriptome sequencing identifies *SPL7*‐regulated copper acquisition genes *FRO4*/*FRO5* and the copper dependence of iron homeostasis in *Arabidopsis* . The Plant Cell, 24, 738–761. 22374396 10.1105/tpc.111.090431 PMC3315244

[pld3245-bib-0006] Boutet, S. C. , Cheung, T. H. , Quach, N. L. , Liu, L. , Prescott, S. L. , Edalati, A. , … Rando, T. A. (2012). Alternative polyadenylation mediates microRNA regulation of muscle stem cell function. Cell Stem Cell, 10, 327–336. 22385659 10.1016/j.stem.2012.01.017 PMC3306803

[pld3245-bib-0007] Browse, J. , & Howe, G. A. (2008). New weapons and a rapid response against insect attack. Plant Physiology, 146, 832–838. 18316637 10.1104/pp.107.115683 PMC2259070

[pld3245-bib-0008] Campo, S. , Peris‐Peris, C. , Siré, C. , Moreno, A. B. , Donarie, L. , Zytnicki, M. , … Segundo, B. S. (2013). Identification of a novel microRNA(miRNA) from rice that targets an alternatively splicing transcript of the *Nramp6* (*Natural resistance‐associated macrophage protein 6*) gene involved in pathogen resistance. New Phytologist, 199, 212–227. 23627500 10.1111/nph.12292

[pld3245-bib-0009] Carretero‐Paulet, L. , Galstyan, A. , Roig‐Villanova, I. , Martínez‐García, J. F. , Bilbao‐Castro, J. R. , & Robertson, D. L. (2010). Genome‐wide classification and evolutionary analysis of the bHLH family of transcription factors in *Arabidopsis*, poplar, rice, moss and algae. Plant Physiology, 153, 1398–1412. 20472752 10.1104/pp.110.153593 PMC2899937

[pld3245-bib-0500] Causier, B. , Ashworth, M. , Guo, W. , & Davies, B. (2012). The TOPLESS interactome: A framework for gene repression in *Arabidopsis* . Plant Physiology, 158, 423‐438. 22065421 10.1104/pp.111.186999 PMC3252085

[pld3245-bib-0010] Chini, A. , Fonseca, S. , Chico, J. M. , Fernández‐Calvo, P. , & Solano, R. (2009). The ZIM domain mediates homo‐ and heteromeric interactions between *Arabidopsis* JAZ proteins. The Plant Journal, 59, 77–87. 19309455 10.1111/j.1365-313X.2009.03852.x

[pld3245-bib-0011] Chini, A. , Fonseca, S. , Fernández, G. , Adie, B. , Chico, J. M. , Lorenzo, O. , … Ponce, M. R. et al (2007). The JAZ family of repressors is the missing link in jasmonate signalling. Nature, 448, 666–671. 17637675 10.1038/nature06006

[pld3245-bib-0012] Chini, A. , Gimenez‐Ibanez, S. , Goossens, A. , & Solano, R. (2016). Redundancy and specificity in jasmonate signalling. Current Opinion in Plant Biology, 33, 147–156. 27490895 10.1016/j.pbi.2016.07.005

[pld3245-bib-0013] Chung, H. S. , Cooke, T. F. , DePew, C. L. , Patel, L. C. , Ogawa, N. , Kobayashi, Y. , & Howe, G. A. (2010). Alternative splicing expands the repertoire of dominant JAZ repressors of jasmonate signaling. The Plant Journal, 63, 613–622. 20525008 10.1111/j.1365-313X.2010.04265.x PMC2966510

[pld3245-bib-0014] Chung, H. S. , & Howe, G. A. (2009). A critical role for the TIFY motif in repression of jasmonate signaling by a stabilized splice variant of the JASMONATE ZIM‐Domain protein JAZ10 in *Arabidopsis* . The Plant Cell, 21, 131–145. 19151223 10.1105/tpc.108.064097 PMC2648087

[pld3245-bib-0015] Cui, Z. , Tong, A. , Huo, Y. , Yan, Z. , Yang, W. , Yang, X. , & Wang, X. X. (2017). SKIP controls flowering time via the alternative splicing of SEF pre‐mRNA in *Arabidopsis* . BMC Biology, 15, 1–17. 28893254 10.1186/s12915-017-0422-2 PMC5594616

[pld3245-bib-0016] Dai, X. , & Zhao, P. X. (2011). psRNATarget: a plant small RNA target analysis server. Nucleic Acids Research, 39, W155–W159. 21622958 10.1093/nar/gkr319 PMC3125753

[pld3245-bib-0017] Feng, G. , Burleigh, J. G. , Braun, E. L. , Mei, W. , & Barbazuk, W. B. (2017). Evolution of the 3R‐MYB gene family in plants. Genome Biology and Evolution, 9, 1013–1029. 28444194 10.1093/gbe/evx056 PMC5405339

[pld3245-bib-0018] Feng, J. , Li, J. , Gao, Z. , Lu, Y. , Yu, J. , Zheng, Q. , … Zhu, Z. (2015). SKIP confers osmotic tolerance during salt stress by controlling alternative gene splicing in *Arabidopsis* . Molecular Plant, 8, 1038–1052. 25617718 10.1016/j.molp.2015.01.011

[pld3245-bib-0019] Foissac, S. , & Sammeth, M. (2007). ASTALAVISTA: dynamic and flexible analysis of alternative splicing events in custom gene datasets. Nucleic Acids Research, 35, W297–W299. 17485470 10.1093/nar/gkm311 PMC1933205

[pld3245-bib-0020] Fonseca, S. , Chini, A. , Hamberg, M. , Adie, B. , Porzel, A. , Kramell, R. , … Solano, R. (2009). (+)‐7‐*iso*‐Jasmonoyl‐L‐isoleucine is the endogenous bioactive jasmonate. Nature Chemical Biology, 5, 344–350. 19349968 10.1038/nchembio.161

[pld3245-bib-0021] Gasperini, D. , Chételat, A. , Acosta, I. F. , Goossens, J. , Pauwels, L. , Goossens, A. , … Farmer, E. E. (2015). Multilayered organization of jasmonate signalling in the regulation of root growth. PLOS Genetics, 11, e1005300. 26070206 10.1371/journal.pgen.1005300 PMC4466561

[pld3245-bib-0600] Haas, B. J. , Papanicolaou, A. , Yassour, M. , Grabherr, M. , Blood P. D. , Bowden J , … Regev, A . (2013). De novo transcript sequence reconstruction from RNA‐seq using the Trinity platform for reference generation and analysis. Nature Protocols, 8(8), 1494–512. 10.1038/nprot.2013.084 23845962 PMC3875132

[pld3245-bib-0022] Haga, N. , Kato, K. , Murase, M. , Araki, S. , Kubo, M. , Demura, T. , … Jürgens, G. et al (2007). R1R2R3‐Myb proteins positively regulate cytokinesis through activation of *KNOLLE* transcription in *Arabidopsis thaliana* . Development, 134, 1101–1110. 17287251 10.1242/dev.02801

[pld3245-bib-0023] Hibara, K. , Isono, M. , Mimura, M. , Sentoku, N. , Kojima, M. , Sakakibara, H. , … Nagato, Y. (2016). Jasmonate regulates juvenile‐to‐adult phase transition in rice. Development, 143, 3407–3416. 27578792 10.1242/dev.138602

[pld3245-bib-0024] Howe, G. A. , & Jander, G. (2008). Plant immunity to insect herbivores. Annual Review of Plant Biology, 59, 41–66. 10.1146/annurev.arplant.59.032607.092825 18031220

[pld3245-bib-0025] Hu, Y. , Jiang, L. , Wang, F. , & Yu, D. (2013). Jasmonate regulates the INDUCER OF CBF EXPRESSION‐C‐REPEAT BINDING FACTOR/DRE BINDING FACTOR1 cascade and freezing tolerance in *Arabidopsis* . The Plant Cell, 25, 2907–2924. 23933884 10.1105/tpc.113.112631 PMC3784588

[pld3245-bib-0026] Jiang, Y. , Liang, G. , Yang, S. , & Yu, D. (2014). *Arabidopsis* WRKY57 functions as a node of convergence for jasmonic acid‐ and auxin‐mediated signaling in jasmonic acid‐induced leaf senescence. The Plant Cell, 26, 230–245. 24424094 10.1105/tpc.113.117838 PMC3963572

[pld3245-bib-0027] Kalsotra, A. , Wang, K. , Li, P. F. , & Cooper, T. A. (2010). MicroRNAs coordinate an alternative splicing network during mouse postnatal heart development. Genes & Development, 24, 653–658. 20299448 10.1101/gad.1894310 PMC2849122

[pld3245-bib-0028] Kalyna, M. , Simpson, C. G. , Syed, N. H. , Lewandowska, D. , Marquez, Y. , Kusenda, B. , … McNicol, J. et al (2012). Alternative splicing and nonsense‐mediated decay modulate expression of important regulatory genes in *Arabidopsis* . Nucleic Acids Research, 40, 2454–2469. 22127866 10.1093/nar/gkr932 PMC3315328

[pld3245-bib-0029] Kato, K. , Gális, I. , Suzuki, S. , Araki, S. , Demura, T. , Criqui, M. C. , … Matsuoka, K. et al (2009). Preferential up‐regulation of G2/M phage‐specific genes by overexpression of the hyperactive form of NtmybA2a lacking its negative regulation domain in tobacco BY‐2 Cells. Plant Physiology, 149, 1945–1957. 19244455 10.1104/pp.109.135582 PMC2663760

[pld3245-bib-0030] Katsir, L. , Chung, H. S. , Koo, A. J. K. , & Howe, G. A. (2008). Jasmonate signaling: a conserved mechanism of hormone sensing. Current Opinion in Plant Biology, 11, 428–435. 18583180 10.1016/j.pbi.2008.05.004 PMC2560989

[pld3245-bib-0031] Kervestin, S. , & Jacobson, A. (2012). NMD: a multifaceted response to premature translational termination. Nature Reviews Molecular Cell Biology, 13, 700–712. 23072888 10.1038/nrm3454 PMC3970730

[pld3245-bib-0032] Koh, J. , Chen, S. , Zhu, N. , Yu, F. , Soltis, P. S. , & Soltis, D. E. (2012). Comparative proteomics of the recently and recurrently formed natural allopolyploid *Tragopogon mirus* (Asteraceae) and its parents. New Phytologist, 196, 292–305. 22861377 10.1111/j.1469-8137.2012.04251.x

[pld3245-bib-0033] Kornblihtt, A. R. , Schor, I. E. , Alló, M. , Dujardin, G. , Petrillo, E. , & Muñoz, M. J. (2013). Alternative splicing: a pivotal step between eukaryotic transcription and translation. Nature Reviews Molecular Cell Biology, 14, 153–165. 23385723 10.1038/nrm3525

[pld3245-bib-0034] Kozomara, A. , & Griffiths‐Jones, S. (2014). miRBase: annotating high confidence microRNAs using deep sequencing data. Nucleic Acids Research, 42, D68–D73. 24275495 10.1093/nar/gkt1181 PMC3965103

[pld3245-bib-0035] Letunic, I. , Doerks, T. , & Bork, P. (2015). SMART: recent updates, new developments and status in 2015. Nucleic Acids Research, 43, D257–D260. 25300481 10.1093/nar/gku949 PMC4384020

[pld3245-bib-0036] Li, J. Y. , Fu, Y. L. , Pike, S. M. , Bao, J. , Tian, W. , Zhang, Y. , … Huang, J. et al (2010). The *Arabidopsis* nitrate transporter NRT1.8 functions in nitrate removal from the xylem sap and mediates cadmium tolerance. The Plant Cell, 22, 1633–1646. 20501909 10.1105/tpc.110.075242 PMC2899866

[pld3245-bib-0037] Li, Y. , Xia, C. , Feng, J. , Yang, D. , Wu, F. , Cao, Y. , … Ma, L. (2016). The SNW domain of SKIP is required for its integration into the spliceosome and its interaction with the Paf1 complex in *Arabidopsis* . Molecular Plant, 9, 1040–1050. 27130079 10.1016/j.molp.2016.04.011

[pld3245-bib-0038] Lim, G. H. , Zhang, X. , Chung, M. S. , Lee, D. J. , Woo, Y. M. , Cheong, H. S. , & Kim, C. S. (2009). A putative novel transcription factor, AtSKIP, is involved in abscisic acid signalling and confers salt and osmotic tolerance in *Arabidopsis* . New Phytologist, 185, 103–113. 19765229 10.1111/j.1469-8137.2009.03032.x

[pld3245-bib-0039] Linkies, A. , & Leubner‐Metzger, G. (2012). Beyond gibberellins and abscisic acid: how ethylene and jasmonates control seed germination. Plant Cell Reports, 31, 253–270. 22044964 10.1007/s00299-011-1180-1

[pld3245-bib-0040] Lu, H. , McChung, R. , & Zhang, C. (2017). Tick tock: Circadian regulation of plant innate immunity. Annual Review of Phytopathology, 55, 287–311. 10.1146/annurev-phyto-080516-035451 28590878

[pld3245-bib-0041] Marchler‐Bauer, A. , Derbyshire, M. K. , Gonzales, N. R. , Lu, S. , Chitsaz, F. , Geer, L. Y. , … Hurwitz, D. I. et al (2015). CDD: NCBI’s conserved domain database. Nucleic Acids Research, 43, D222–D226. 25414356 10.1093/nar/gku1221 PMC4383992

[pld3245-bib-0042] Meng, Y. , Shao, C. , Ma, X. , & Wang, H. (2013). Introns targeted by plant microRNAs: a possible novel mechanism of gene regulation. Rice, 6, 8. 24280590 10.1186/1939-8433-6-8 PMC4883735

[pld3245-bib-0043] Meyer, K. , Koester, T. , & Staiger, D. (2015). Pre‐mRNA splicing in plants: *in vivo* functions of RNA‐binding proteins implicated in the splicing process. Biomolecules, 5, 1717–1714. 26213982 10.3390/biom5031717 PMC4598772

[pld3245-bib-0044] Miao, Y. , & Zentgraf, U. (2007). The antagonist function of *Arabidopsis* WRKY53 and ESR/ESP in leaf senescence is modulated by the jasmonic and salicylic acid equilibrium. The Plant Cell, 19, 819–830. 17369373 10.1105/tpc.106.042705 PMC1867371

[pld3245-bib-0045] Monte, I. , Franco‐Zorrilla, J. M. , García‐Casado, G. , Zamarreño, A. M. , García‐Mina, J. M. , Nishihama, R. , … Solano, R. (2019). A single JAZ repressor controls the jasmonate pathway in *Marchantia polymorpha* . Molecular Plant, 12, 185–198. 30594656 10.1016/j.molp.2018.12.017

[pld3245-bib-0046] Moreno, J. E. , Shyu, C. , Campos, M. L. , Patel, L. C. , Chung, H. S. , Yao, J. , … Howe, G. A. (2013). Negative feedback control of jasmonate signaling by an alternative splice variant of JAZ10. Plant Physiology, 162, 1006–1017. 23632853 10.1104/pp.113.218164 PMC3668036

[pld3245-bib-0047] Pauwels, L. , Barbero, G. F. , Geerinck, J. , Tilleman, S. , Grunewald, W. , Pérez, A. C. , … Gil, E. et al (2010). NINJA connects the co‐repressor TOPLESS to jasmonate signalling. Nature, 464, 788–791. 20360743 10.1038/nature08854 PMC2849182

[pld3245-bib-0048] Perez‐Santángelo, S. , Mancini, E. , Francey, L. J. , Schlaen, R. G. , Chernomoretz, A. , Hogenesch, J. B. , & Yanovsky, M. J. (2014). Role for LSM genes in the regulation of circadian rhythms. Proceedings of the National Academy of Sciences of the United States of America, 111, 15166–15171. 25288739 10.1073/pnas.1409791111 PMC4210300

[pld3245-bib-0049] Pfaff, C. , Ehrnsberger, H. F. , Flores‐Tornero, M. , Sørensen, B. B. , Schubert, T. , Längst, G. , … Grasser, K. D. (2018). ALY RNA‐binding proteins are required for nucleocytosolic mRNA transport and modulate plant growth and development. Plant Physiology, 177, 226–240. 29540591 10.1104/pp.18.00173 PMC5933122

[pld3245-bib-0050] Qi, T. , Huang, H. , Song, S. , & Xie, D. (2015). Regulation of jasmonate‐mediated stamen development and seed production by a bHLH‐MYB complex in *Arabidopsis* . The Plant Cell, 27, 1620–1633. 26002869 10.1105/tpc.15.00116 PMC4498206

[pld3245-bib-0051] Qi, T. , Wang, J. , Huang, H. , Liu, B. , Gao, H. , Liu, Y. , … Xie, D. (2015). Regulation of jasmonate‐induced leaf senescence by antagonism between bHLH subgroup IIIe and IIId factors in *Arabidopsis* . The Plant Cell, 27, 1634–1649. 26071420 10.1105/tpc.15.00110 PMC4498205

[pld3245-bib-0052] Reddy, A. S. N. , Marquez, Y. , Kalyna, M. , & Barta, A. (2013). Complexity of the alternative splicing landscape in plants. The Plant Cell, 25, 3657–3683. 24179125 10.1105/tpc.113.117523 PMC3877793

[pld3245-bib-0053] Sandberg, R. , Neilson, J. R. , Sarma, A. , Sharp, P. A. , & Burge, C. B. (2008). Proliferating cells express mRNAs with shortened 3’ untranslated regions and fewer microRNA target sites. Science, 320, 1643–1647. 18566288 10.1126/science.1155390 PMC2587246

[pld3245-bib-0054] Seo, M. , Aoki, H. , Koiwai, H. , Kamiya, Y. , Nambara, E. , & Koshiba, T. (2004). Comparative studies on the *Arabidopsis* aldehyde oxidase (AAO) gene family revealed a major role of AAO3 in ABA biosynthesis in seeds. Plant and Cell Physiology, 45, 1694–1793. 15574845 10.1093/pcp/pch198

[pld3245-bib-0055] Shannon, P. , Markiel, A. , Ozier, O. , Baliga, N. S. , Wang, J. T. , Ramage, D. , … Ideker, T. (2003). Cytoscape: A software environment for integrated models of biomolecular interaction networks. Genome Research, 13, 2498–2504. 14597658 10.1101/gr.1239303 PMC403769

[pld3245-bib-0056] Shilov, I. V. , Seymour, S. L. , Patel, A. A. , Loboda, A. , Tang, W. H. , Keating, S. P. , … Schaeffer, D. A. (2007). The paragon algorithm, a next generation search engine that uses sequence temperature values and feature probabilities to identify peptides from tandem mass spectra. Molecular & Cellular Proteomics, 6, 1638–1655. 17533153 10.1074/mcp.T600050-MCP200

[pld3245-bib-0057] Shyu, C. , Figueroa, P. , DePew, C. L. , Cooke, T. F. , Sheard, L. B. , Moreno, J. E. , … Howe, G. A. (2012). JAZ8 lacks a canonical degron and has an EAR motif that mediates transcriptional repression of jasmonate responses in *Arabidopsis* . The Plant Cell, 24, 536–550. 22327740 10.1105/tpc.111.093005 PMC3315231

[pld3245-bib-0058] Sikder, H. A. , Devlin, M. K. , Dunlap, S. , Ryu, B. , & Alani, R. M. (2003). Id proteins in cell growth and tumorigenesis. Cancer Cell, 3, 525–530. 12842081 10.1016/s1535-6108(03)00141-7

[pld3245-bib-0059] Song, S. , Qi, T. , Huang, H. , Ren, Q. , Wu, D. , Chang, C. , … Xie, D. (2011). The Jasmonate‐ZIM domain proteins interact with the R2R3‐MYB transcription factors MYB21 and MYB24 to affect jasmonate‐regulated stamen development in *Arabidopsis* . The Plant Cell, 23, 1000–1013. 21447791 10.1105/tpc.111.083089 PMC3082250

[pld3245-bib-0060] Staiger, D. , & Brown, J. W. S. (2013). Alternative splicing at the intersection of biological timing, development, and stress responses. The Plant Cell, 25, 3640–3656. 24179132 10.1105/tpc.113.113803 PMC3877812

[pld3245-bib-0061] Storozhenko, S. , Inzé, D. , Montagu, M. V. , & Kushnir, S. (2001). *Arabidopsis* coactivator ALY‐like proteins, DIP1 and DIP2 interact physically with the DNA‐binding domain of the Zn‐finger poly(ADP‐ribose) polymerase. Journal of Experimental Botany, 52, 1375–1380. 11432957

[pld3245-bib-0062] Szklarczyk, D. , Franceschini, A. , Wyder, S. , Forslund, K. , Heller, D. , Huerta‐Cepas, J. , … Tsafou, K. P. et al (2015). STRING V10: protein‐protein interaction networks, integrated over the tree of life. Nucleic Acids Research, 43, D447–D452. 25352553 10.1093/nar/gku1003 PMC4383874

[pld3245-bib-0063] Tanaka, H. , Maruta, T. , Ogawa, T. , Tanabe, N. , Tamoi, M. , Yoshimura, K. , & Shigeoka, S. (2015). Identification and characterization of *Arabidopsis* AtNUDX9 as a GDP‐D‐mannose pyrophosphohydrolase: its involvement in root growth inhibition in response to ammonium. Journal of Experimental Botany, 66, 5797–5808. 26049160 10.1093/jxb/erv281 PMC4566977

[pld3245-bib-0064] Thatcher, L. F. , Cevik, V. , Grant, M. , Zhai, B. , Jones, J. D. G. , Manners, J. M. , & Kazan, K. (2016). Characterization of a *JAZ7* activation‐tagged *Arabidopsis* mutant with increased susceptibility to the fungal pathogen *Fusarium oxysporum* . Journal of Experimental Botany, 67, 2367–2386. 26896849 10.1093/jxb/erw040 PMC4809290

[pld3245-bib-0065] Thatcher, S. R. , Danilevskaya, O. N. , Meng, X. , Beatty, M. , Zastrow‐Hayes, G. , Harris, C. , … Li, B. (2016). Genome‐wide analysis of alternative splicing during development and drought stress in maize. Plant Physiology, 170, 586–599. 26582726 10.1104/pp.15.01267 PMC4704579

[pld3245-bib-0066] Thines, B. , Katsir, L. , Melotto, M. , Niu, Y. , Mandaokar, A. , Liu, G. , … Browse, J. (2007). JAZ repressor proteins are targets of the SCF^COI1^ complex during jasmonate signalling. Nature, 448, 661–665. 17637677 10.1038/nature05960

[pld3245-bib-0067] Thireault, C. , Shyu, C. , Yoshida, Y. , Aubin, B. S. , Campos, M. L. , & Howe, G. A. (2015). Repression of jasmonate signaling by a non‐TIFY JAZ protein in *Arabidopsis* . The Plant Journal, 82, 669–679. 25846245 10.1111/tpj.12841

[pld3245-bib-0068] Trapnell, C. , Hendrickson, D. G. , Sauvageau, M. , Goff, L. , Rinn, J. L. , & Pachter, L. (2013). Differential analysis of gene regulation at transcript resolution with RNA‐seq. Nature Biotechnology, 31, 46–53. 10.1038/nbt.2450 PMC3869392 23222703

[pld3245-bib-0069] Valenzuela, C. E. , Acevedo‐Acevedo, O. , Miranda, G. S. , Vergara‐Barros, P. , Holuigue, L. , Figueroa, C. R. , & Figueroa, P. M. (2016). Salt stress response triggers activation of the jasmonate signaling pathway leading to inhibition of cell elongation in *Arabidopsis* primary root. Journal of Experimental Botany, 67, 4209–4220. 27217545 10.1093/jxb/erw202 PMC5301928

[pld3245-bib-0070] Voinnet, O. (2009). Origin, biogenesis, and activity of plant microRNAs. Cell, 136, 669–687. 19239888 10.1016/j.cell.2009.01.046

[pld3245-bib-0071] Wang, L. , Wang, S. , & Li, W. (2012). RSeQC: quality control of RNA‐seq experiments. Bioinformatics, 28, 2184–2185. 22743226 10.1093/bioinformatics/bts356

[pld3245-bib-0072] Wang, X. , Wu, F. , Xie, Q. , Wang, Y. , Yue, Y. , Gahura, O. , … Ma, L. (2012). SKIP is a component of the spliceosome linking alternative splicing and the circadian clock in *Arabidopsis* . The Plant Cell, 24, 3278–3295. 22942380 10.1105/tpc.112.100081 PMC3462631

[pld3245-bib-0073] Wu, G. , & Poethig, R. S. (2006). Temporal regulation of shoot development in *Arabidopsis thaliana* by *miR156* and its target *SPL3* . Development, 133, 3539–3547. 16914499 10.1242/dev.02521 PMC1610107

[pld3245-bib-0074] Wu, T. D. , & Nacu, S. (2010). Fast and SNP‐tolerant detection of complex variants and splicing in short reads. Bioinformatics, 26, 873–881. 20147302 10.1093/bioinformatics/btq057 PMC2844994

[pld3245-bib-0075] Yamasaki, H. , Hayashi, M. , Fukazawa, M. , Kobayashi, Y. , & Shikanai, T. (2009). SQUAMOSA promoter binding protein‐like7 is a central regulator for copper homeostasis in *Arabidopsis* . The Plant Cell, 21, 347–361. 19122104 10.1105/tpc.108.060137 PMC2648088

[pld3245-bib-0076] Yan, H. , Yoo, M. J. , Koh, J. , Liu, L. , Chen, Y. , Acikgoz, D. , … Chen, S. (2014). Molecular reprogramming of *Arabidopsis* in response to perturbation of jasmonate signaling. Journal of Proteome Research, 13, 5751–5766. 25311705 10.1021/pr500739v

[pld3245-bib-0077] Yan, Y. , Stolz, S. , Chételat, A. , Reymond, P. , Pagni, M. , Dubugnon, L. , & Farmer, E. (2007). A downstream mediator in the growth repression limb of the jasmonate pathway. The Plant Cell, 19, 2470–2483. 17675405 10.1105/tpc.107.050708 PMC2002611

[pld3245-bib-0078] Yang, X. , Zhang, H. , & Li, L. (2012). Alternative mRNA processing increases the complexity of microRNA‐based gene regulation in *Arabidopsis* . The Plant Journal, 70, 421–431. 22247970 10.1111/j.1365-313X.2011.04882.x

[pld3245-bib-0079] Yu, J. , Zhang, Y. , Di, C. , Zhang, Q. , Zhang, K. , Wang, C. , … Yuan, J. S. et al (2016). JAZ7 negatively regulates dark‐induced leaf senescence in *Arabidopsis* . Journal of Experimental Botany, 67, 751–762. 26547795 10.1093/jxb/erv487 PMC4737072

[pld3245-bib-0080] Zhai, Q. , Zhang, X. , Wu, F. , Feng, H. , Deng, L. , Xu, L. , … Li, C. (2015). Transcriptional mechanism of jasmonate receptor COI1‐mediated delay of flowering time in *Arabidopsis* . The Plant Cell, 27, 2814–2828. 26410299 10.1105/tpc.15.00619 PMC4682329

[pld3245-bib-0081] Zhang, F. , Ke, J. , Zhang, L. , Chen, R. , Sugimoto, K. , Howe, G. A. , … Melcher, K. (2017). Structural insights into alternative splicing‐mediated desensitization of jasmonate signaling. Proceedings of the National Academy of Sciences of the United States of USA, 114, 1720–1725. 10.1073/pnas.1616938114 PMC5320967 28137867

[pld3245-bib-0082] Zhang, R. , Calixto, C. P. G. , Marquez, Y. , Venhuizen, P. , Tzioutziou, N. A. , Guo, W. , … Ten Have, S. et al (2017). A high quality *Arabidopsis* transcriptome for accurate transcript‐level analysis of alternative splicing. Nucleic Acids Research, 45, 5061–5073. 28402429 10.1093/nar/gkx267 PMC5435985

[pld3245-bib-0083] Zhang, X. , Min, J. H. , Huang, P. , Chung, J. S. , Lee, K. H. , & Kim, C. S. (2014). AtSKIP functions as a mediator between cytokinin and light signaling pathway in *Arabidopsis thaliana* . Plant Cell Reports, 33, 401–409. 24258244 10.1007/s00299-013-1540-0

[pld3245-bib-0084] Zheng, Y. , Cui, X. , Su, L. , Fang, S. , Chu, J. , Gong, Q. , … Zhu, Z. (2017). Jasmonate inhibits COP1 activity to suppress hypocotyl elongation and promote cotyledon opening in etiolated *Arabidopsis* seedlings. The Plant Journal, 90, 1144–1155. 28321936 10.1111/tpj.13539

