# Supplementary material for: Jasmonate induced alternative splicing responses in Arabidopsis
Source: Plant Direct. 2020 Aug 26;4(8):e00245. doi: 10.1002/pld3.245 (PMC7450174; doi:10.1002/pld3.245)
Supplement: Supplementary file 3 — Supplementary Material [file PLD3-4-e00245-s003.pdf]

***The Plant Journal* Supporting Information**

Article title: Jasmonate Induced Alternative Splicing Responses in Arabidopsis

Authors: Guanqiao Feng, Mi-Jeong Yoo, Ruth Davenport, J. Lucas Boatwright, Jin Koh, Sixue Chen, W. Brad Barbazuk*

The following supporting information is available for this article:

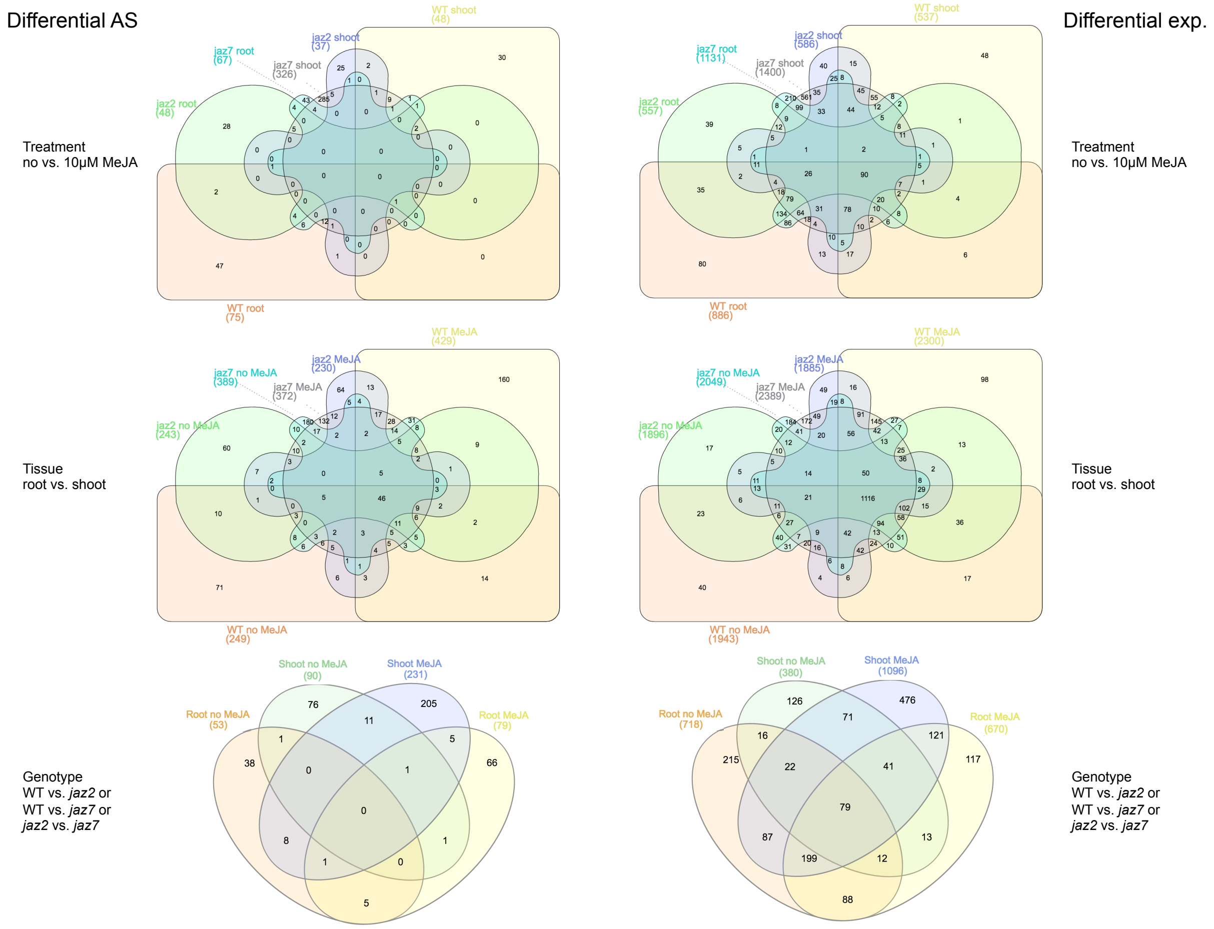


**Figure S1.** Venn diagram of genes that exhibit differential expression or differential AS in response to treatment (MeJA), between tissues (root and shoots) and between mutant backgrounds.

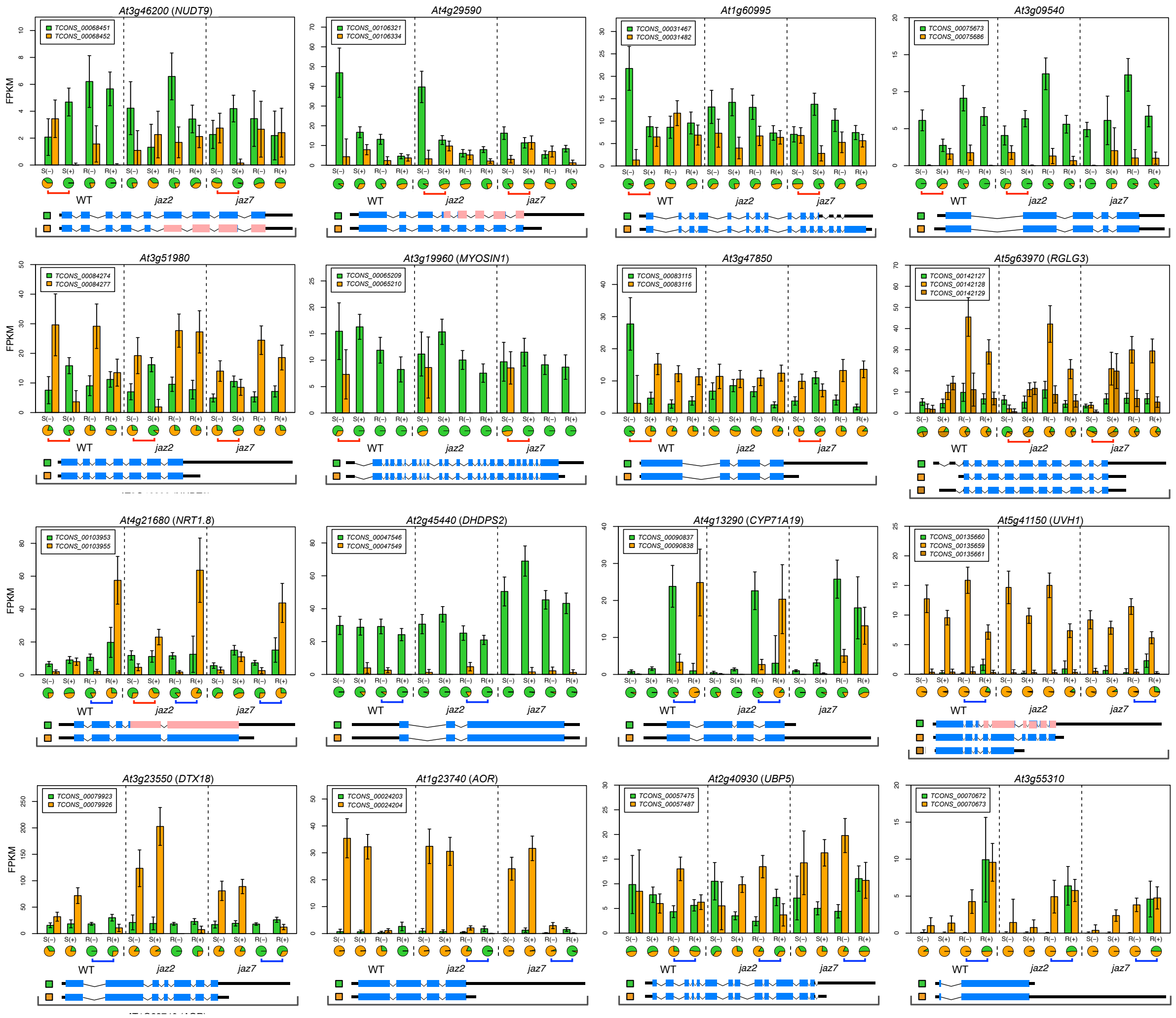

**Figure S2.** Example cases in which gene expression was regulated by AS in response to MeJA treatment. Pie charts under each condition indicate the proportion of each AS isoform present relative to the total expression from the locus. Red and blue lines under pies indicate significant differential AS in shoot and root, respectively. Under barplot are the isoform structures of the gene. Hat lines indicate introns; black thin boxes indicate UTR; blue boxes indicate CDS; pink boxes indicate regions which were turned to non-coding regions due to AS caused PTC.

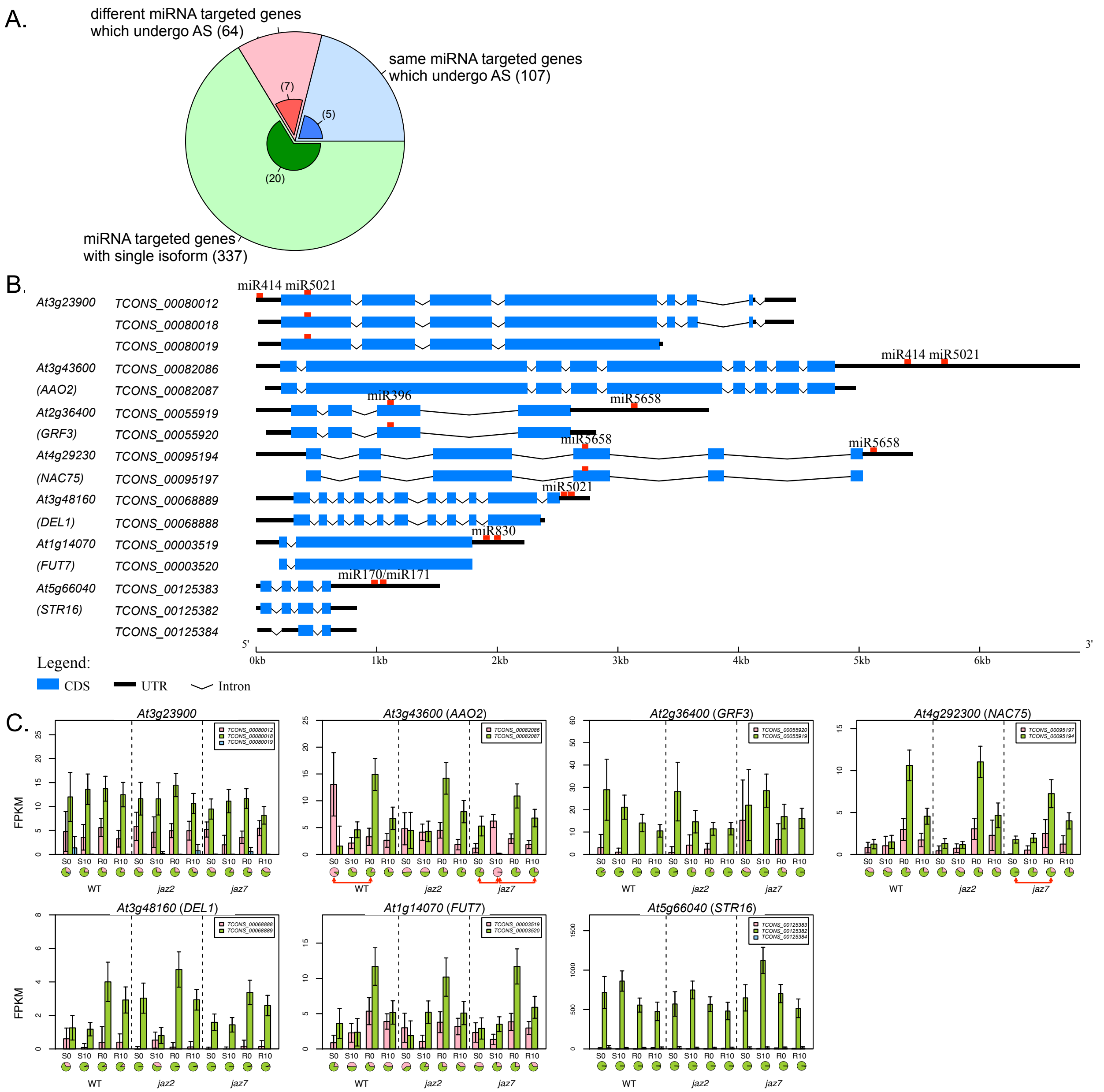


**Figure S3.** Genes undergoing AS that include miRNA binding sites that differ between isoforms. A) Distribution of predicted miRNA target sites in genes that do not undergo AS, genes that undergo AS and AS isoforms of the gene contain different miRNA target sites and genes that undergo AS to produce isoforms with the same miRNA target sites. The darker shaded sections within each category indicate cases with multiple miRNA target sites. B) Gene structure of the seven cases (dark pink in A) with the predicted miRNA binding site indicated. C) Expression profiles of the seven genes shown in B. Pie charts under each condition indicate the proportion of

each AS isoform relative to the total expression from the locus. Significantly differential AS is indicated by red lines.

| miRNA | No. | Gene | Transcript | Target | Target site |
|---|---|---|---|---|---|
| ath-miR156 | 1 | *AT1G35515* (*MYB8*/*HOS10*) | *TCONS_00008685* | 0 | |
| | | | *TCONS_00008686* | 1 | ath-miR156 [798-817: CUUCUCUCUCUCUUCUCUCA] |
| ath-miR172 | 2 | *AT3G54990* (*SMZ*) | *TCONS_00085221* | 1 | ath-miR172 [965-985: UUGCAGCAUCAUCAGGAUUCC] |
| | | | *TCONS_00085222* | 0 | |
| ath-miR414 | 3 | *AT3G43600* (*AAO2*) | *TCONS_00082086* | 2 | ath-miR414 [4777-4797: UGAUGAUGAUGAUGAAGAUGC]<br>ath-miR5021 [5090-5109: UUUUCUUCUUCUUCUUCUUC] |
| | | | *TCONS_00082087* | 0 | |
| | 4 | *AT2G23140* (*PUB4*) | *TCONS_00051815* | 1 | ath-miR414 [2818-2838: GGAUGAUGAUGAUGAUGAUGA] |
| | | | *TCONS_00051822* | 1 | ath-miR414 [2877-2897: GGAUGAUGAUGAUGAUGAUGA] |
| | | | *TCONS_00051834* | 0 | |
| | 5 | *AT5G47040* (*LON2*/*APEM10*) | *TCONS_00137229* | 0 | |
| | | | *TCONS_00137255* | 1 | ath-miR414 [916-939: UGACAACGAUGAUGAUGAAGAUGA] |
| ath-miR415 | 6 | *AT4G12560* (*CPR1*/*CPR30*) | *TCONS_00090637* | 1 | ath-miR415 [1441-1461: CUUUUCUGUCUCUGCUCUGUU] |
| | | | *TCONS_00090640* | 0 | |
| | | | *TCONS_00090641* | 1 | ath-miR415 [1396-1416: CUUUUCUGUCUCUGCUCUGUU] |
| ath-miR472 | 7 | *AT5G43730* (*RSG2*) | *TCONS_00119400* | 0 | |
| | | | *TCONS_00119402* | 1 | ath-miR472 [1313-1334: GGUAUGGGGGGAAUAGGAAAAA] |
| | | | *TCONS_00119403* | 1 | ath-miR472 [1313-1334: GGUAUGGGGGGAAUAGGAAAAA] |
| | | | *TCONS_00119404* | 0 | |
| | 8 | *AT1G15885* | *TCONS_00003955* | 0 | |
| | | | *TCONS_00003956* | 1 | ath-miR472 [1191-1212: GGUAUGGGGGGAGUAGGUAAAA] |
| ath-miR5021 | 9 | *AT4G19670* | *TCONS_00103441* | 1 | ath-miR5021 [47-67: UUCUUCUUCUUCUUCUUCUCU] |
| | | | *TCONS_00103442* | 1 | ath-miR5021 [47-67: UUCUUCUUCUUCUUCUUCUCU] |
| | | | *TCONS_00103445* | 0 | |
| | | | *TCONS_00103446* | 1 | ath-miR5021 [44-64: UUCUUCUUCUUCUUCUUCUCU] |
| | 10 | *AT2G27030* (*CAM5*) | *TCONS_00042668* | 0 | |
| | | | *TCONS_00042669* | 0 | |
| | | | *TCONS_00042670* | 1 | ath-miR5021 [24-44: UUCUUCUUCUUCUUCUUCUCU] |
| | 11 | *AT2G46340* (*SPA1*) | *TCONS_00059096* | 0 | |
| | | | *TCONS_00059100* | 1 | ath-miR5021 [128-147: CUUUCUUCUUCUUCUUCUUC] |
| | 12 | *AT3G02740* | *TCONS_00060511* | 1 | ath-miR5021 [114-133: UUUUCUUCUUCUUCUUCUUU] |
| | | | *TCONS_00060512* | 0 | |
| | 13 | *AT3G05180* | *TCONS_00074206* | 1 | ath-miR5021 [1620-1640: UUCUUCUUCUUCUUCUUCUCU] |
| | | | *TCONS_00074207* | 0 | |
| | 14 | *AT3G44610* | *TCONS_00082295* | 1 | ath-miR5021 [18-38: UUCUUCUUCUUCUUCUUCUCU] |
| | | | *TCONS_00082304* | 0 | |
| | 15 | *AT4G13930* (*SHM4*) | *TCONS_00101323* | 1 | ath-miR5021 [2480-2500: UUUAUCUUCUUCUUCUUCUCC] |
| | | | *TCONS_00101325* | 0 | |
| | 16 | *AT5G60860* (*RABA1F*) | *TCONS_00124016* | 1 | ath-miR5021 [57-76: UUUUUUUGUUCUUCUUCUCA] |
| | | | *TCONS_00124018* | 0 | |
| ath-miR5641 | 17 | *AT5G55530* | *TCONS_00122678* | 0 | |
| | | | *TCONS_00122680* | 0 | |
| | | | *TCONS_00122682* | 0 | |
| | | | *TCONS_00122683* | 1 | ath-miR5641 [60-80: UCUUUCUAUCAUCUUCUUACA] |
| | 18 | *AT5G44650* (*CEST*/*Y3IP1*) | *TCONS_00119648* | 1 | ath-miR5641 [1180-1200: UUAUUUAAUCAUCUUCUUCCU] |
| | | | *TCONS_00119653* | 1 | ath-miR5641 [1180-1200: UUAUUUAAUCAUCUUCUUCCU] |
| | | | *TCONS_00119656* | 0 | |
| ath-miR5658 | 19 | *AT5G64330* (*NPH3*/*RPT3*) | *TCONS_00124898* | 0 | |
| | | | *TCONS_00124899* | 0 | |
| | | | *TCONS_00124902* | 1 | ath-miR5658 [3646-3666: AAUCAUCAUCAUAAUCAUCAU] |
| | 20 | *AT4G29230* (*NAC75*) | *TCONS_00095194* | 2 | ath-miR5658 [1487-1507: GAUCAUCACCAUCAUCAUCAU]<br>ath-miR5658 [1981-2001: GCUCAUCGUCAUCAUCAUCAU] |
| | | | *TCONS_00095197* | 1 | ath-miR5658 [1078-1098: GAUCAUCACCAUCAUCAUCAU] |
| ath-miR8177 | 21 | *AT1G18880* (*NPF2.9*) | *TCONS_00004939* | 0 | |
| | | | *TCONS_00004941* | 1 | ath-miR8177 [3771-3791: UA-GAGUGACACAUCAUCACAA] |

AS.diff — Treatment: a shoot_WT; b shoot_*jaz2*; c shoot_*jaz7*; d root_WT; e root_*jaz2*; f root_*jaz7*

AS.diff — Tissue: a No MeJA_WT; b No MeJA_*jaz2*; c No MeJA_*jaz7*; d 10um MeJA_WT; e 10um MeJA_*jaz2*; f 10um MeJA_*jaz7*

AS.diff — Genotype: a *jaz2*-WT(shoot_no MeJA); b *jaz2*-WT(shoot_10um MeJA); c *jaz2*-WT(root_no MeJA); d *jaz2*-WT(root_10um MeJA); e *jaz7*-WT(shoot_no MeJA); f *jaz7*-WT(shoot_10um MeJA); g *jaz7*-WT(root_no MeJA); h *jaz7*-WT(root_10um MeJA); i *jaz2*-*jaz7*(shoot_no MeJA); j *jaz2*-*jaz7*(shoot_10um MeJA); k *jaz2*-*jaz7*(root_no MeJA); l *jaz2*-*jaz7*(root_10um MeJA)

**Figure S4.** Twenty-one genes which contain miRNA binding sites potentially subjected to AS regulation. Blue, green and yellow shading indicate significantly changed isoform proportions identified in treatment, tissue or genotype comparisons, respectively. Targeted sequences within the transcript and their locations are presented within brackets [ ] in the "Target site" column.

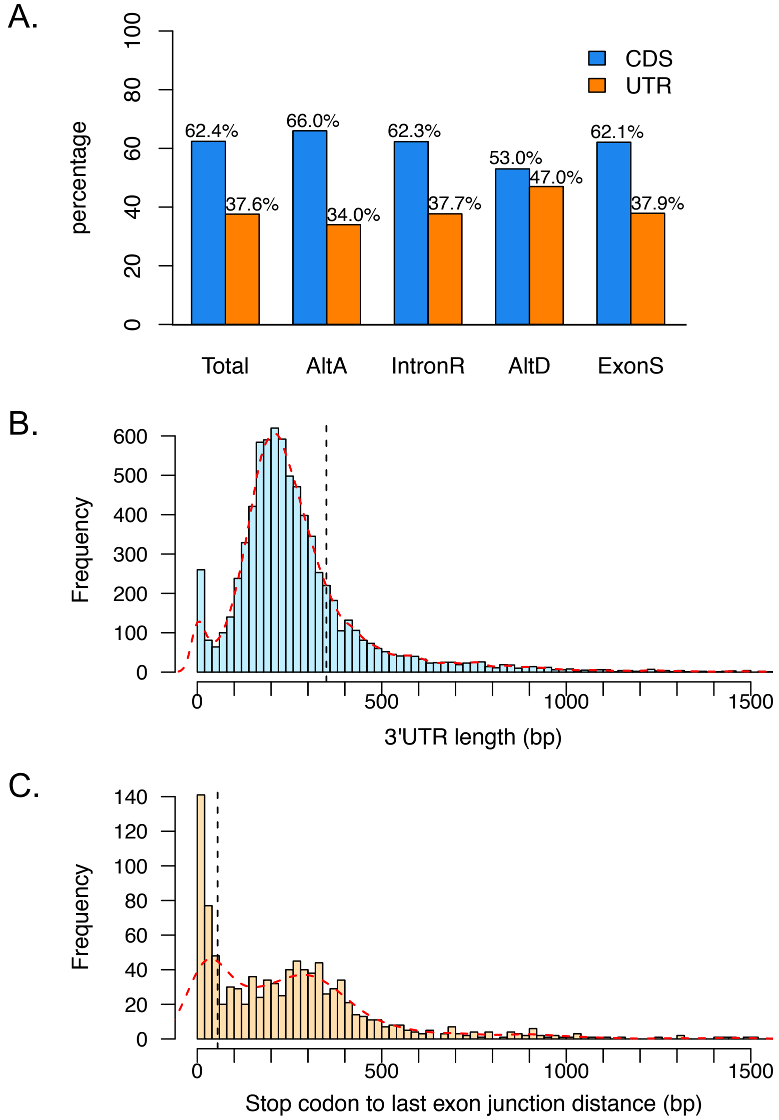


**Figure S5.** Cases in which AS won't generate multiple protein products. A) Distribution of AS events occurring within the CDS or UTRs. B) Distribution of 3'-UTR lengths of all identified transcripts. C) Distribution of the distance between the stop codon and the last exon junction when the stop codon is not within the last exon of the transcript.

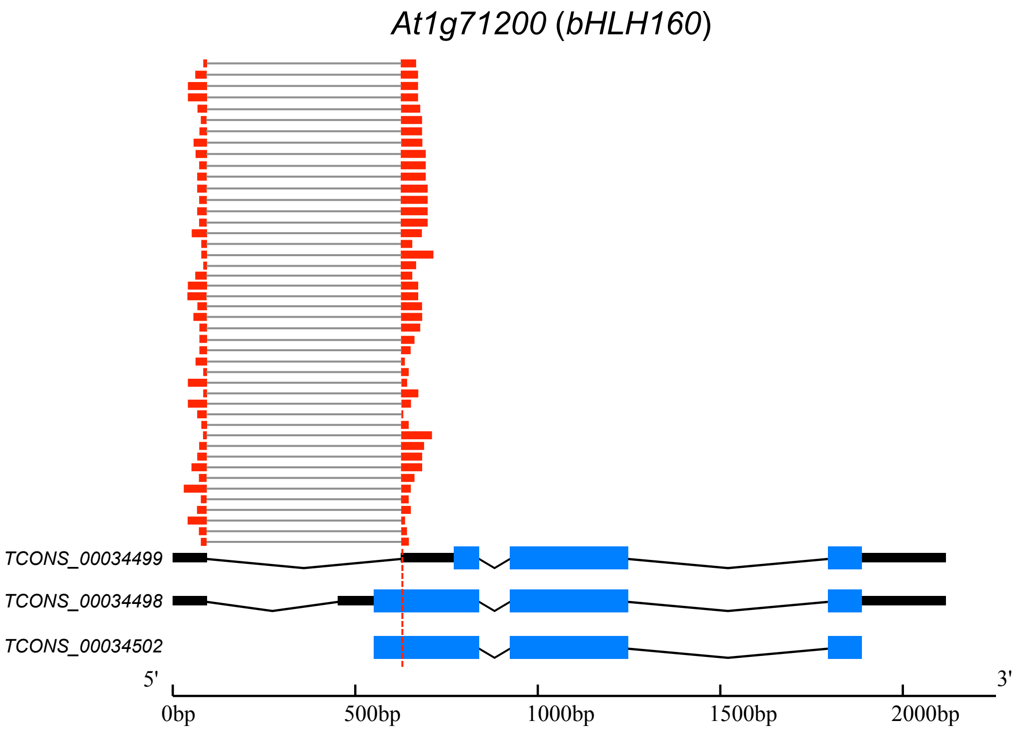


**Figure S6.** Mapped reads from the MeJA project supporting the AS junction in *Arabidopsis bHLH160*$^{b-}$. Hat lines indicate introns; black thin boxes indicate UTR; blue boxes indicate CDS. Gray line connected red boxes indicate mapped reads supporting the novel junction in the alternative isoform *TCONS_00034499*.

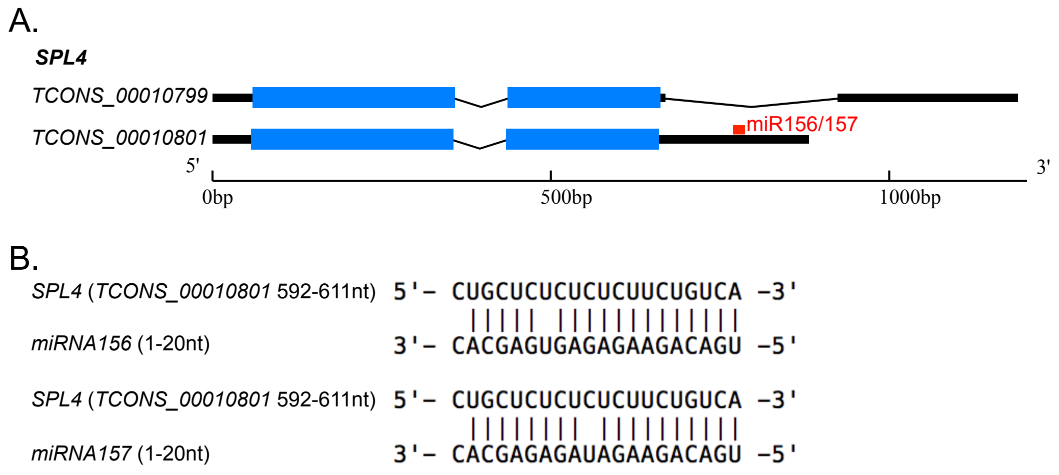


**Figure S7.** Differential miRNA regulation of *SPL4* splice variants. A) Gene structure of *SPL4* AS isoforms with the predicted miRNA binding site indicated. B) Complementarity of the miRNA with the predicted *SPL4* target site.

**Table S1.** RNA-seq library and mapping information.

| Samples | # Raw reads | # Mapped reads | Percentage |
|---|---|---|---|
| WT_shoot_0_rep1 | 21,128,469 | 17,025,286 | 80.6% |
| WT_shoot_0_rep2 | 14,186,832 | 8,909,077 | 62.8% |
| WT_shoot_0_rep3 | 23,486,963 | 8,769,393 | 37.3% |
| WT_shoot_10_rep1 | 13,657,112 | 10,920,597 | 80.0% |
| WT_shoot_10_rep2 | 16,134,503 | 12,685,395 | 78.6% |
| WT_shoot_10_rep3 | 13,510,226 | 10,641,409 | 78.8% |
| WT_root_0_rep1 | 15,241,432 | 12,494,869 | 82.0% |
| WT_root_0_rep2 | 40,886,447 | 32,315,025 | 79.0% |
| WT_root_0_rep3 | 22,960,991 | 18,992,644 | 82.7% |
| WT_root_10_rep1 | 15,351,676 | 13,060,948 | 85.1% |
| WT_root_10_rep2 | 17,683,061 | 14,960,418 | 84.6% |
| WT_root_10_rep3 | 15,102,434 | 12,796,169 | 84.7% |
| jaz2_shoot_0_rep1 | 15,032,167 | 10,950,279 | 72.8% |
| jaz2_shoot_0_rep2 | 11,958,237 | 9,261,665 | 77.5% |
| jaz2_shoot_0_rep3 | 9,277,191 | 5,794,838 | 62.5% |
| jaz2_shoot_10_rep1 | 11,154,705 | 8,481,602 | 76.0% |
| jaz2_shoot_10_rep2 | 11,431,445 | 8,887,788 | 77.7% |
| jaz2_shoot_10_rep3 | 11,715,590 | 9,311,499 | 79.5% |
| jaz2_root_0_rep1 | 15,850,717 | 13,107,489 | 82.7% |
| jaz2_root_0_rep2 | 17,974,716 | 15,159,325 | 84.3% |
| jaz2_root_0_rep3 | 17,514,424 | 14,196,519 | 81.1% |
| jaz2_root_10_rep1 | 16,661,319 | 13,609,917 | 81.7% |
| jaz2_root_10_rep2 | 23,840,265 | 19,031,731 | 79.8% |
| jaz2_root_10_rep3 | 18,466,668 | 15,379,759 | 83.3% |
| jaz7_shoot_0_rep1 | 10,507,564 | 8,446,985 | 80.4% |
| jaz7_shoot_0_rep2 | 10,564,614 | 8,097,926 | 76.7% |
| jaz7_shoot_0_rep3 | n.a. | n.a. | n.a. |
| jaz7_shoot_10_rep1 | 12,594,778 | 9,115,023 | 72.4% |

To be continue

| Samples | # Raw reads | # Mapped reads | Percentage |
|---|---|---|---|
| jaz7_shoot_10_rep2 | 17,867,067 | 13,564,544 | 75.9% |
| jaz7_shoot_10_rep3 | 15,161,488 | 11,884,760 | 78.4% |
| jaz7_root_0_rep1 | 17,388,880 | 14,375,611 | 82.7% |
| jaz7_root_0_rep2 | 17,181,387 | 14,247,505 | 82.9% |
| jaz7_root_0_rep3 | 15,300,499 | 13,159,910 | 86.0% |
| jaz7_root_10_rep1 | 16,636,604 | 13,859,228 | 83.3% |
| jaz7_root_10_rep2 | 13,528,403 | 11,442,447 | 84.6% |
| jaz7_root_10_rep3 | 20,662,422 | 17,233,780 | 83.4% |
| Total | 577,601,296 | 452,171,360 | 78.3% |
